# Multiepitope-Based Subunit Vaccine Design and Evaluation against Respiratory Syncytial Virus Using Reverse Vaccinology Approach

**DOI:** 10.3390/vaccines8020288

**Published:** 2020-06-08

**Authors:** Muhammad Tahir ul Qamar, Zeeshan Shokat, Iqra Muneer, Usman Ali Ashfaq, Hamna Javed, Farooq Anwar, Amna Bari, Barira Zahid, Nazamid Saari

**Affiliations:** 1 College of Life Science and Technology, Guangxi University, Nanning 530004, China; m.tahirulqamar@hotmail.com; 2 Department of Bioinformatics and Biotechnology, Government College University, Faisalabad 38000, Pakistan; meharzeeshan5274@gmail.com (Z.S.); usmancemb@gmail.com (U.A.A.); hamnaj203@gmail.com (H.J.); 3 School of Life Sciences, University of Science and Technology of China, Hefei 230052, China; iqra@mail.ustc.edu.cn; 4 Department of Chemistry, University of Sargodha, Sargodha 40100, Pakistan; fqanwar@yahoo.com; 5 Hubei Key Laboratory of Agricultural Bioinformatics, College of Informatics, Huazhong Agricultural University, Wuhan 430070, China; amnabari99@yahoo.com; 6 Key Laboratory of Horticultural Plant Biology (Ministry of Education), Huazhong Agricultural University, Wuhan 430070, China; barirazahid@outlook.com; 7 Department of Food Science, Faculty of Food Science and Technology, Universiti Putra Malaysia, Serdang 43400, Selangor, Malaysia

**Keywords:** respiratory disorders, respiratory syncytial virus, multiepitope-based vaccine, Subunit vaccine, computational approaches

## Abstract

Respiratory syncytial virus (RSV) is primarily associated with respiratory disorders globally. Despite the availability of information, there is still no competitive vaccine available for RSV. Therefore, the present study has been designed to develop a multiepitope-based subunit vaccine (MEV) using a reverse vaccinology approach to curb RSV infections. Briefly, two highly antigenic and conserved proteins of RSV (glycoprotein and fusion protein) were selected and potential epitopes of different categories (B-cell and T-cell) were identified from them. Eminently antigenic and overlapping epitopes, which demonstrated strong associations with their respective human leukocyte antigen (HLA) alleles and depicted collective ~70% coverage of the world’s populace, were shortlisted. Finally, 282 amino acids long MEV construct was established by connecting 13 major histocompatibility complex (MHC) class-I with two MHC class-II epitopes with appropriate adjuvant and linkers. Adjuvant and linkers were added to increase the immunogenic stimulation of the MEV. Developed MEV was stable, soluble, non-allergenic, non-toxic, flexible and highly antigenic. Furthermore, molecular docking and molecular dynamics (MD) simulations analyses were carried out. Results have shown a firm and robust binding affinity of MEV with human pathogenic toll-like receptor three (TLR3). The computationally mediated immune response of MEV demonstrated increased interferon-γ production, a significant abundance of immunoglobulin and activation of macrophages which are essential for immune-response against RSV. Moreover, MEV codons were optimized and in silico cloning was performed, to ensure its increased expression. These outcomes proposed that the MEV developed in this study will be a significant candidate against RSV to control and prevent RSV-related disorders if further investigated experimentally.

## 1. Introduction

Respiratory syncytial virus (RSV) belongs to the RNA virus family with a genome size of 15.19 Kb (ID: 5145) that is negative, single-stranded, non-segmented, and enveloped [1]. It belongs to the family *Paramyxoviridae* and has been categorized into the genus *Orthopnemovirus* [1]. RSV has been categorized in 2 subtypes that further include several strains: RSV-A (13 strains: GA1—GA7, SAA1, NA1—NA4 and ON1), and RSV-B (22 strains: GB1—GB4, SAB1—SAB4, URU1—URU2, BA1—BA10, BA—C and THB) [2,3]. Regarding its number of genes and proteins, it is considered to be the most complex virus of the family. It is also distinctly different from other family members. The RSV virion has a nucleocapsid wrapped in a lipid cover that is mainly produced by the host cells’ plasma membrane [4]. The genome of RSV consists of 10 genes of which two are non-structural proteins: NS1 and NS2. Others include phospho-protein (P), nucleoprotein (N), matric protein (M), M2, small hydrophobic protein (SH), fusion protein (F), glycoprotein (G) and large polymerase (L). The G, F, and SH proteins are envelope proteins. The P, N, M, M2, and L proteins are present just below the envelope [4,5]. The virus binds to the host cell surface using F-protein which later directs its entry into the cell leading towards syncytia formation [6]. The surface glycoproteins (G and F) facilitate the synthesis of host-neutralizing antibodies and are thus involved in pathogenesis [4]. In 1955, RSV was first isolated from a chimpanzee band, and shortly after that, it was found in children with respiratory disorders. It was observed that the pathogenic agent is highly contagious [7]. The mode of RSV transmission is mainly droplets or direct exposure to the infected person. When an infected person coughs or sneezes, RSV in droplets form can enter other persons’ nose, eyes or mouth, and causes infection. It also spreads if a healthy person touches the surface that has RSV (https://www.cdc.gov/rsv/about/transmission.html). Infections are recurrent because RSV disturbs the long-term immunologic memory. The most vulnerable victims of RSV are infants. RSV, mainly responsible for upper respiratory tract infections (URTIs), may also cause lower respiratory tract infections (LRTIs in the form of bronchiolitis) [8]. Symptoms are much like the common cold as purulent discharge primarily nasal, sore throat, fever and blockage of air canals due to mucus. However, severe infection may cause complications leading towards respiratory disorders including asthma, bronchiolitis, and pneumonia. RSV’s cytopathic effect is far less as compared to its other prototypes, so it can be inferred that the damage done to air canals is more of an immune response rather than the viral lysis of the host cells [9].

Despite knowing so much about the RSV genome, replication, and epidemiology, there is still a lack of competitive vaccines. Several vaccines are under clinical trials. One of the vaccines that have shown to be promising is developed by Novavax, Inc. Rockville, Maryland, USA. It is an F-protein targeting vaccine with an aluminum phosphate adjuvant, called RSV-F vaccine [10]. Major challenges to develop a vaccine against RSV are: (i) early age RSV infection especially in neonates and infants, when the immune system is immature; (ii) RSV infection in elderly people (>65 years), when the immune system is compromised; (iii) RSV multiple mechanisms of innate immune system invasion; (iv) failure of humoral immune response that induce immunity to thwarts RSV reinfection; (v) mutations in RSV genome; (vi) vaccine associated boosted illness, and (vii) absence of suitable animal models for testing [11,12,13]. This century has seen a remarkable advancement in vaccine development techniques aided by bioinformatics and immunoinformatics. Techniques like reverse vaccinology and structural vaccinology have boosted the rate of viral vaccine development [14]. Protein antigenicity can be predicted to a high degree of accuracy [15]. To prepare a feasible subunit vaccine, different antigenic determinants must be selected, and adjuvants should be added to increase efficiency. It will help to nudge the immune-system and improve immune responses in the host [16]. Prediction of potential epitopes and the development of multiepitope-based subunit vaccine (MEV) construct that could trigger cell-mediated as well as humoral immunity response, becomes a new footpath with technology and bioinformatics advances [16,17,18,19,20,21,22]. Besides, Sub-unit vaccines have the significant potential to overcome challenges associated with RSV vaccine development as, they can be used for maternal immunization, they established from different antigenic epitopes thus they have rare chance to cause vaccine-enhanced illness, and they are also effective for elderly immunization [13,23,24,25,26,27]. 

**Figure 1:**
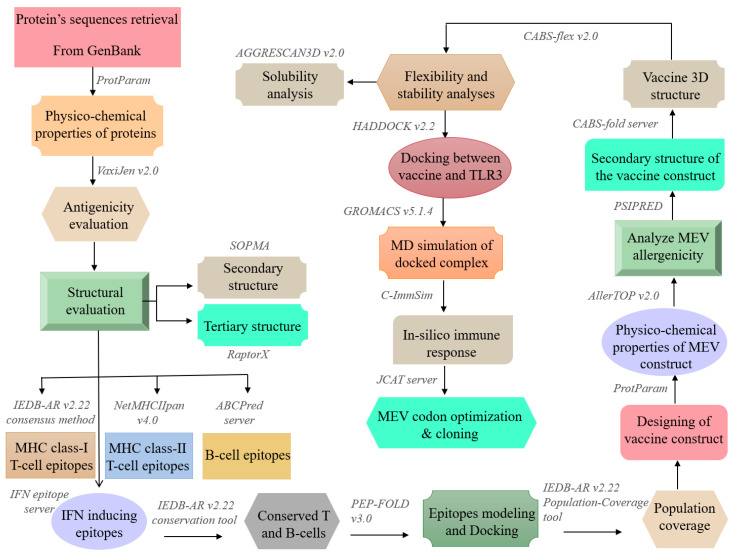
A schematic representation of methodology and tools used in the present study.

In the current study, we aimed to use a set of immunoinformatics tools to design an MEV against RSV. A visual summary of the general workflow is given in Figure 1. RSV G protein is the primary target of the humoral immune response, and in animal models, G protein targeting antibodies revealed to neutralize RSV and to provide protection against severe RSV infection [28]. RSV F protein is highly conserved and is the only RSV membrane protein that is essential for its infection, and considered as the most relevant target for antiviral therapeutic strategies [29]. Therefore, G and F proteins were selected and used to forecast B- and T-cell epitopes that could induce IFN-γ, followed by MEV-construction and homology-modeling. The vaccine protein was exposed to thorough evaluations including immunological and physicochemical suitability, stability, flexibility, solubility and binding affinity towards the TLR3 immune receptor and the stimulation of likely immune responses. Finally, in silico cloning with optimized codons was carried out to enhance the vaccine protei’s translation efficiency.

## 2. Materials and Methods

### 2.1. Immunoinformatic Analyses of the Antigen

#### 2.1.1. Proteins Sequence Retrieval, Antigenicity Prediction, Physicochemical Properties, and Structural Evaluation

First, RSV G and F proteins sequences were retrieved in FASTA format from GENBANK [30]. Then, ProtParam tool of the ExPASy server (https://web.expasy.org/protparam/ ) [31] was used to assess the physical and chemical properties of the selected proteins. ProtParam computes the physico-chemical properties from protein sequence, however it cannot specify post-translational modifications of protein, and also can not specify either mature protein will form monomer or multi-dimer. The protein antigenicity was determined using the VaxiJen v.2.0 tool (http://www.ddg-pharmfac.net/vaxijen/VaxiJen/VaxiJen.html) [15]. It computes the properties based on alignment-free method and mainly from physico-chemical properties. The secondary structure analysis was performed through SOPMA (https://npsa-prabi.ibcp.fr/cgi-bin/npsa_automat.pl?page=/NPSA/npsa_sopma.html) [32]. Tertiary structures of the final stage of most of the RSV proteins are not reported yet. Therefore, RaptorX [33] was used for the tertiary structure modeling of target proteins. RaptorX (http://raptorx.uchicago.edu/) is among the widely used structure prediction tools, that uses homology-based approach to predicted 3D structure of query protein. The GalaxyRefine2 server was utilized to improve the predicted 3D proteins models. Finally, using Rampage Server (http://mordred.bioc.cam.ac.uk/~rapper/rampage.php), predicted models were analyzed for Ramachandran plot to verify the quality and accuracy of predicted models.

#### 2.1.2. Prediction and Assessment of T-Cell Epitopes

First, the immune epitope database analysis resource (IEDB-AR) v.2.22 (http://tools.iedb.org/main/) [34] consensus method was exploited to assess the 12-mer MHC class-I T-cell epitopes. IEDB-AR is an online repository that provides several tools for prediction and analyses of anti-genic epitopes (http://tools.immuneepitope.org/main/). The FASTA format sequences of the amino acids were given, and all available alleles were preferred to predict epitopes of T-cells. Epitopes with a consensus score less than two were thought to be excellent binders and were selected for further analysis. Next, using all of the alleles, 15-mer MHC class-II T-cell epitopes were obtained using the latest NetMHCIIpan 4.0 server (http://www.cbs.dtu.dk/services/NetMHCIIpan/ ) [35]. The epitopes were classified at the default threshold as strong, weak and non-binding based on the percentage value. Strong binding, weak binding, and non-binding percentile criteria were 2%, 10%, and higher than 10%, respectively. Additionally, the assessment of immunogenicity, antigenicity, allergenicity, and toxicity were performed for each epitope. The VaxiJen v.2.0 server and IEDB-AR v.2.22 MHC-I immunogenicity tool were used to assess the antigenicity and immunogenicity [36], respectively. Moreover, allergenic profiling was done by using AllergenFP v.1.0 server (http://ddg-pharmfac.net/AllergenFP/) [37] and toxicity prediction by the ToxinPred server (http://crdd.osdd.net/raghava/toxinpred/) [38]. The descriptor-based alignment-free fingerprint method was applied by the above-mentioned servers to make ensure 88.9% prediction precision [37] meanwhile to predict the toxicity of peptides, the last server makes use of machine-learning with quantitative matrix [38].

#### 2.1.3. Prediction and Assessment of B-Cell Epitopes

Epitopes of B-cell are critical components of the immune system that trigger an adaptive immune response and could, therefore, be used as crucial vaccine building blocks. These are of two categories, conformational and linear B-cell epitopes [39]. ABCPred server (http://crdd.osdd.net/raghava/abcpred/) was utilized to forecast the 16-mer linear B-cell epitopes [40]. ABCPred server uses neural networking based approach to forecast linear epitopes (http://www.imtech.res.in/raghava/abcpred/). The Minimum value to forecast the B-cell epitope was set 0.5 in the ABCPred tool. Furthermore, to forecast the conformational B-cell epitopes, the ElliPro integrated tool (http://tools.iedb.org/ellipro/) [41] provided by the IEDB-AR v.2.22 was used. ElliPro does not require training data-sets and works based on geometrical properties of protein structure (http://tools.iedb.org/ellipro/). Additionally, the identified B-cell epitopes were evaluated by VaxiJen v.2.0, ToxinPred and AllergenFP v.1.0 servers for their antigenic, toxic, and allergic profiles respectively.

#### 2.1.4. Conservation Analysis and Shortlisting of Predicted Epitopes

To select the best-conserved T- and B-cell epitopes, conservation analysis was done using the IEDB-AR v.2.22 epitope conservancy analysis tool [42]. Epitopes with 100% conservation were selected for further study. To sort out the effectual epitopes, cytokine-prompting capabilities were viewed as an important parameter. IFN-γ is acknowledged for intrinsic safe responses and can confine viral duplication right away [43,44]. Moreover, they can trigger the flexible immune reactions by preparing cytotoxic T lymphocyte (CTL) and Helper T lymphocyte (HTL). The IFN epitope server (http://crdd.osdd.net/raghava/ifnepitope/) was used to calculate the IFN-γ-inducing potential of forecasted epitopes utilizing support vector machine (SVM) hybrid and MERCI algorithms [45]. Finally, those epitopes which passed through toxicity, conservancy, overlapping, and antigenicity tests, were further checked for their resemblance to human proteins using jackHMMER with an e-value of 1 × 10^−10^ and similarity <75% parameters [46]. In resemblance checking, epitopes with similarity above 75% with any part of the human proteome were removed. 

#### 2.1.5. Epitopes Modeling and Molecular Docking

Molecular docking of the epitopes binding to specific human leukocytes antigen (HLA) alleles was performed to evaluate their binding effectiveness. Concisely, selected HTL and CTL epitopes were submitted to PEP-FOLD v.3.0 server (https://bioserv.rpbs.univ-paris-diderot.fr/services/PEP-FOLD3/), for de-novo structure prediction, utilizing the sOPEP sorting scheme with 200 simulations [47]. This structure prediction server was constructed to forecast the conformations of linear peptides (5–50 amino acids) depending on the taboo sampling/forward backtrack algorithm [47]. The protein structural data of HLA alleles were obtained from the RCSB Protein Data Bank (PDB) [48], and those HLA alleles without a crystal structure were modeled using comparative homology approaches. Molecular docking was carried out using the same procedure of our studies published beforehand [20,21,45]. The PyMOL molecular graphic system v.1.3 (https://pymol.org/) was utilized to draw and visualize the docked complex [49].

#### 2.1.6. Assessment of Population Coverage 

The HLA alleles have different levels of dispersion and expression as indicated by the ethnicities and areas all over the world [50,51], hence, impact the development of an effective vaccine [42,52]. The selected epitopes were submitted to the IEDB-AR v.2.22 Population Coverage tool [42] to compute the population coverage percentages [42]. This tool calculated the population coverage of every epitope for various geographical areas dependent on the distribution of HLA-binding alleles. Consequently, chosen epitopes and their specific HLA-binding alleles (MHC class-I and II) were examined. In the current study, we highlighted the areas of specific significance with the RSV pathogen.

### 2.2. Multiple-Epitope Vaccines Designing and Evaluation 

#### 2.2.1. Designing of Vaccine Construct

To construct a subunit vaccine, the epitopes with following properties are usually preferred: (i) highly antigenic/non-allergic, (ii) 100% conserved, (iii) overlapping, (iv) with significant population coverage, (v) having a strong binding affinity with common human allele, and (vi) have no similarity with the human proteins. Therefore, only those epitopes were further selected that following the above parameters to construct MEV. To boost the immune response an adjuvant was attached with the first CTL epitope with the EAAAK linker, while other epitopes were connected using AAY and GPGPG linkers to conserve their independent immunogenic activities after their inter-interaction compatibility validation. The β-defensin adjuvant was used in the present study, because it is a thoroughly basic 45 amino acids long peptide that behaves both as an antimicrobial agent and as an immunomodulator [53].

#### 2.2.2. Primary and Secondary Structural Analyses

First, Blastp analysis [54] was executed against *Homo sapiens* proteome with default parameters (threshold: 10; word size: 6; matrix BLOSUM62) to validate that the designed MEV sequence is non-homologous. Protein with less than 37% identity generally considered as non-homologous protein. Next, ProtParam was used to assess the physicochemical properties of the MEV construct [55]. It predicts various physicochemical properties such as (Instability index, Grand average hydropathy, aliphatic index, half-life, and theoretical isoelectric point (PI)) depending on the amino acids approximations involved in the pk [56]. AllerTOP v.2.0 server (https://www.ddg-pharmfac.net/AllerTOP/) was used to analyze MEV allergenicity and non-allergic nature [57]. According to guidelines with 2210 non-allergens of alike species and 2210 allergens of distinct species, it uses the data protein-sequence by putting K nearest neighbor calculation (kNN; k = 3). To predict the antigenicity of the vaccine, the Vaxijen v.2.0 server was used. The secondary structure of the vaccine construct was determined by using the PSIPRED workbench (http://bioinf.cs.ucl.ac.uk/psipred/) [58]. This test also evaluated a different number of vaccines building properties such as extended chain, random coil, alpha helices, and degree of beta-turns.

#### 2.2.3. Tertiary Structure Prediction, Refinement and Validation

As the designed vaccine is a collection of different epitopes and no suitable template was available: therefore, the 3D structure of MEV was determined using the CABS-fold server de novo modeling approach (http://biocomp.chem.uw.edu.pl/CABSfold/). This server is based on CABS modeling approach, combined with multiscale modeling pipeline and Replica Exchange Monte Carlo scheme [59]. The GalaxyRefine2 server molecular dynamics (MD) simulation approach was utilized to modify the predicted MEV 3D structure [60]. To confirm the refined MEV structure quality, Ramachandran plot analysis was performed using the RAMPAGE server (http://mordred.bioc.cam.ac.uk/~rapper/rampage.php) [61], followed by structural validation analysis using the ProSA-web server (https://prosa.services.came.sbg.ac.at/prosa.php) [62]. The ERRAT server (https://servicesn.mbi.ucla.edu/ERRAT/) was further utilized to assess the calculation of non-bonded connections in the MEV construct [63]. Furthermore, MEV structural flexibility was also analyzed using CABS-Flex v.2.0 server [64]. The flexibility of the vaccine is an important aspect of its functioning, and the CABS-Flex server provides a detailed overview of the flexibility and stability of query protein by simulating its residues [65].

#### 2.2.4. Screening for B-Cell Epitopes 

Ellipro-tool was used provided by IEDB-AR v.2.22, to forecast the conformational B-cell epitopes for the final MEV construct, utilizing default settings (minimum score: 0.5; maximum distance: 6 Å) [41]. It forecasts epitopes by estimating residual protrusion index (PI), protein shape and neighbor residue clustering [41]. Linear B-cell epitopes were forecasted using the iBCE-EL server (http://www.thegleelab.org/iBCE-EL/) [66]. This server forecast 12 mer Linear B-cell epitopes by default, utilizing a novel gathering learning structure consisting of two independent predictors, i.e., especially randomized-tree and gradient boosting-classifiers [66].

#### 2.2.5. Molecular Docking between Vaccine and TLR3

The host produces an efficient immune response if an antigen/vaccine interacts properly with the target immune cells. Therefore, molecular docking analysis was carried out to examine binding between the MEV and the human immune receptors. TLR3 has been extensively studied and researchers found its vital roles in the generation of an antiviral immune response. HADDOCK v.2.2 (http://haddock.science.uu.nl/enmr/services/HADDOCK2.2/) was used for the MEV docking with TLR3 (PDB ID: 1ZIW). To visualize the docked complex and draw figures, the PyMOL molecular graphic system v.1.3 was used [49]. Besides, the online database PDBsum was used to demonstrate the interacting residues of docked complexes [67].

#### 2.2.6. Molecular Dynamics Simulation Analysis 

MD simulation is an important technique in analyzing the strength of the receptor–ligand complex [52,68]. The TLR3-MEV complex was simulated for 20 ns time using GROMACS v.5.1.4 (http://manual.gromacs.org/documentation/5.1.4/index.html) [69] by following the same protocol of our previously published studies [45,70,71,72,73]. The trajectories were saved for each complex after every 2 fs, the root-mean-square deviation (RMSD), Hydrogen bond analysis, and root-mean-square fluctuations (RMSF) analyses were performed using gmx hbond, gmx rmsd, and gmx rmsf module of GROMACS, respectively.

#### 2.2.7. MM/PBSA Binding Free Energy Calculation

MM/PBSA has been extensively used to estimate binding free energies of protein-ligand systems [74,75,76]. G mmpbsa module of GROMACS v.5.1.4 was used to estimate the binding energy of the simulated TLR3-MEV complex [77]. Total 500 snapshots from the 20 ns simulation trajectory were extracted for the calculation of MM/PBSA. The binding free energy of the protein with ligand in solvent was calculated using the following equation:

(1) $$\Delta G_{binding}=G_{complex}-\left(G_{protein}+G_{ligand}\right)$$

The free energy $G_{x}$ of each term of Equation (1) was calculated using the following equation:

(2) $$G_{x}=<E_{MM}>-TS+<G_{solvation}>$$

where $G_{x}$ is the calculated average free energy and $E_{MM}$ is the average molecular mechanical energy. $E_{MM}$ Includes energy of bonded and nonbonded interactions. $E_{MM}$ was calculated based on the molecular mechanics (MM) force-field parameters. $G_{solvation}$ is the free energy of solvation.

(3) $$E_{MM}=E_{bonded}+E_{nonbonded}$$

$E_{nonbonded}$ Includes both $E_{vdw\;}and\;E_{ele}$ interactions. In the MM-PBSA approach, the solvation free energy was calculated using an implicit solvent model. The solvation free energy was calculated using the following two terms:

(4) $$G_{solvation}=G_{polar}+G_{nonpolar}$$

In Equation (4), $G_{polar}\;and\;G_{nonpolar}$ are electrostatic and non-electrostatic terms respectively.

### 2.3. Immunogenicity Evaluation of the Vaccine Construct

An in silico immune simulation was performed using C-ImmSim server (http://150.146.2.1/C-IMMSIM/index.php) [78], in order to validate the immunological response of constructed MEV. This server simulates the three major functional mammal system components (bone marrow, thymus and lymph node) [78]. The MEV has been tested for the ability to simulate various types of immune cells such as HTL, CTL, NK cells, B-cells, dendritic cells, Immunoglobulins, and cytokines. Clinically the minimum recommended interval between two doses of vaccines is four weeks [52,79,80,81]. Immune simulation was performed using the similar protocol reported by earlier studies [51,52,80,82,83]. Briefly, three injections were administered with the recommended intervals of four weeks (1, 84 and 168 time-steps parameters were set, as one time-step is equal to eight hours of real life) for a total of 1050 steps of simulation. Other parameters were kept as default. Each injection contained default 1000 units of MEV, to estimate MEV-induce active cellular regulations against suitable dose. 

### 2.4. Codon Optimization and In Silico Cloning

Codon adaptation is a method of increasing the translation efficacy of external genes in the host, if the use of codon in both species varies. After careful evaluation of MEV properties and immune response, its codon optimization was performed followed by in silico cloning. For MEV codon optimization, the Java Codon Adaptation Tool (JCAT) server [84] was used to make it compliant with the widely used prokaryotic expression system; *E. coli* K12 [85]. The available extra options were chosen to evade: (a) rho-independent transcription termination, (b) prokaryote ribosome binding-site and (c) restriction enzymes cleavage-sites. The GC (guanine and cytosine) contents together with the codon adaptation index (CAI) [86] were evaluated. Furthermore, to facilitate restriction and cloning, sticky ends restriction sites of HindIII and BamHI restriction enzymes were added at the start/N-terminal and end/C-terminal of the modified MEV sequence, respectively. Furthermore, by using the SnapGene tool, (https:/snapgene.com/) the adapted nucleotide sequence of MEV was cloned into the *E. coli* pET30a (+) vector, to ensure its in vitro expression.

### 2.5. Data Availability 

Supplementary data has been referred in the main manuscript. By utilizing corresponding accession numbers, sequences and structures of the proteins used in the current study can be retrieved from the NCBI and RCSB PDB database.

## 3. Results

### 3.1. Pre-Vaccine Design Analyses

#### 3.1.1. Target Proteins Sequence and Structural Analyses 

The amino acid sequences of RSV G [GENBANK CAA83900.1] and F [GENBANK: BAA00105.1] proteins were retrieved from GENBANK in FASTA format sequence. VaxiJen v.2.0 was then used for checking antigenicity. According to the results, both proteins were significantly antigenic with values 0.5791 and 0.5173 respectively. Besides, secondary structure evaluation and other physicochemical properties such as stability profiling, half-life, theoretical pI, molecular weight, and aliphatic index were also analyzed (Tables S1 and S2). The 3D models of selected proteins were predicted using the RaptorX [87] server and refined with the GalaxyRefining2 tool (Figure S1). The predicted structures quality was evaluated by Ramachandran plot analysis, shown in Table 1. Both predicted models were of the best quality as they demonstrated most of the amino acids within the authorized region. There were two reasons to design these models. Initially, for the mapping of epitopes on the particular protein models, to recognize and affirm their interfaces. Furthermore, to forecast conformational B-cell epitopes utilizing the ElliPro server.

#### 3.1.2. Evaluation and Selection of Epitope

IEDB-AR v.2.22 consensus network servers were used to predict CTL epitopes, and the NetMHCIIpan 4.0 server was used to predict the HTL epitopes. ABCPred server was used for the prediction of linear epitopes of B-cells, and Ellipro server was used to determine conformational B-cell epitopes. The SVM hybrid algorithms excluding Motif were used to predict IFN-γ inducing potential of the predicted epitopes by the IFN epitope server. The criteria designed for selecting the best possible epitopes was that their conservation among the proteins should be 100%, should have the best binding affinity, they should not overlap with the human proteins, significantly antigenic/immunogenic and should not lie inside the post-translational-modification sites and glycosylation-sites of the particular proteins. According to all these specifications, some promising epitopes were recognized in the current study. Total 43 CTL epitopes (G 19 and F 24), 13 HTL epitopes (G 7 and F 6), 51 linear B-cell epitopes (G-20 and F 31) and 12 conformational B-cell epitopes (G 3 and F 9) were identified (Tables S3–S6). 

#### 3.1.3. Molecular Docking between Epitopes and HLA Alleles

To construct a subunit vaccine, the chosen epitopes should be 100% conserved, overlapping and antigenic. Therefore, a total of 15 (13 MHC I and 2 MHC II) overlapping, conserved and highly antigenic epitopes were selected and their binding affinity with their respective HLA alleles was determined utilizing the molecular docking approach (Table 2). 

**Figure 2:**
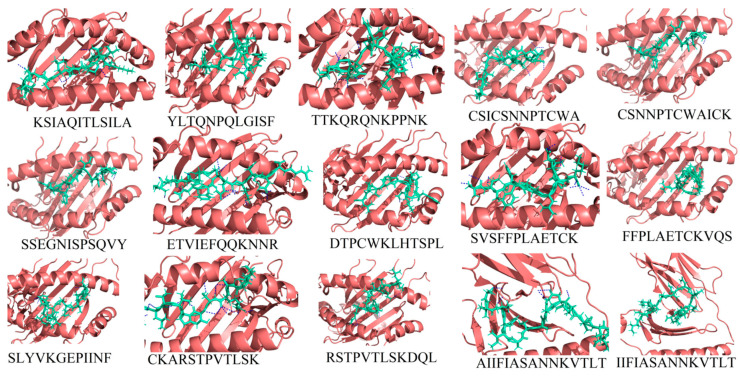
The 3D binding pattern of the selected 15 epitopes (deep-salmon cartoon representation) docked with their respective HLAs (green-cyan sticks representation) as shown in Table 2. Hydrogen bond interactions are highlighted with blue color dotted lines.

It was found that all selected epitopes (Figure S2) bind deep inside in their respective alleles’ binding pockets (Figure 2). Each bound epitope depicted stronger than −10.00 kcal/mol docking affinity and strong hydrogen binding. All the selected epitopes showed significant binding efficacy and also their appropriateness to be utilized in a multiepitope-based subunit vaccine construct.

#### 3.1.4. Population Coverage Analysis

**Figure 3:**
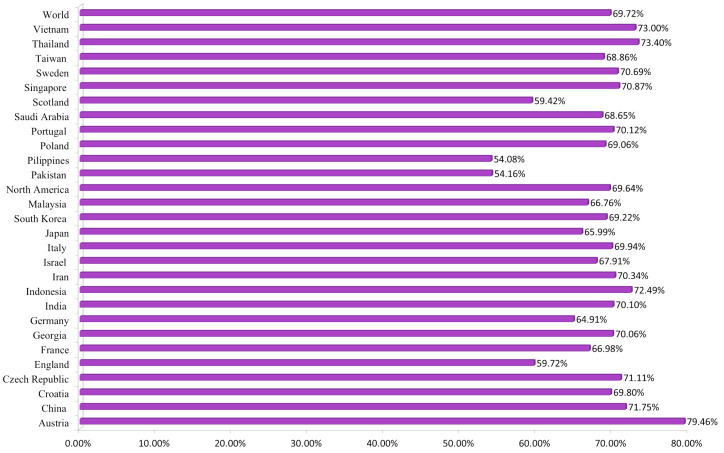
Collective worldwide population coverage of MEV epitopes based on their respective HLA alleles.

The population coverage was collectively calculated for selected 13 CTL and 2 HTL epitopes with their relative HLA alleles. HLA alleles diversify according to world ethnic groups and areas. Therefore, it affects the development of an epitope-based vaccine. Analysis revealed combined coverage of ~70% of the world’s population for selected epitopes (Figure 3). The highest coverage was found within the population of Austria. However, the least population coverage was reported for the Philippines. Our analyses revealed that the population coverage was higher where RSV cases have been investigated before, and selected epitopes would be promising candidates to be used in the MEV construct.

### 3.2. Multiepitope Based Sub-Unit Vaccine Design and Validation

#### 3.2.1. Construction of MEV

**Figure 4:**
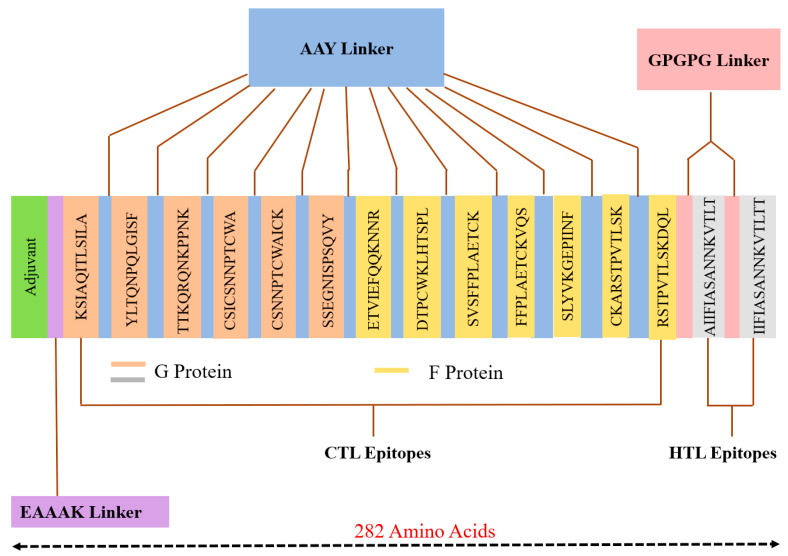
MEV construct overview. It has 282 amino acids, consisting of an adjuvant (green) linked at N-terminal of MEV with the help of EAAAK linker (purple). AAY linker (blue) used to join the CTL epitopes and GPGPG linker (pink) was used to join the HTL epitopes.

All 15 selected epitopes (G 8 and F 7) were further used to develop an MEV construct. An adjuvant (45 amino acid long β-defensin) was bound with the aid of the EAAAK linker at the start (to the MEV N-terminal). EAAAK linker reduces connection with other protein areas with efficient detachment and increases stability [88,89]. The vaccine’s immunogenicity may increase with an adjuvant. Epitopes were merged together based on their interaction’s compatibility in a sequential manner with AAY and GPGPG linkers, respectively. AAY and GPGPG prevent the production of junctional epitopes, that is the main task in the construction of multiepitope vaccines; on the other hand, they promote the immunization and epitope presentation [90,91]. The final vaccine construct consisted of 282 amino acids (Figure 4). 

#### 3.2.2. Physicochemical and Immunogenic Profiling 

First, Blastp analysis [54] was executed against *Homo sapiens* proteome, and results showed MEV has no similarity (higher or equal to 37%) with any human protein. Next, allergenicity, antigenicity and toxicity of the vaccine construct were evaluated. Results described that MEV is highly antigenic (0.6027 at 0.5% threshold), non-allergenic and non-toxic. Furthermore, ProtParam was used to evaluate the physicochemical properties of the MEV construct. The theoretical PI and vaccine molecular weight were 9.48 kDa and 30537.22 kDa, respectively. The mean half-life of the construct was calculated as 30 h in vitro, >20 h in vivo and >10 h in yeast. The estimate of the grand-average-hydropathicity (GRAVY) was −0.122; the negative sign in the score shows the hydrophilic nature of the MEV. The above results indicated MEV as a potential vaccine candidate.

#### 3.2.3. Secondary Structure Analysis

To analyze the secondary structure of MEV, PSIPRED had been used. According to the findings, 74 amino acids participated in the development of α-helix consisting of 26.24% of the overall sequence, for β-strands formation 25 amino acids which are 8.87% and for coils formation 102 amino acid which constitute 36.17% of the entire vaccine construction participated (Figure S3).

#### 3.2.4. Tertiary Structure Prediction, Refinement and Validation 

**Figure 5:**
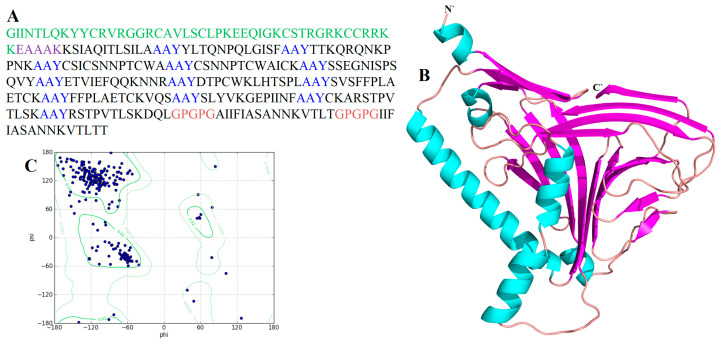
Three-dimensional (3D) structure prediction and validation of MEV construct: (**A**) MEV construct sequence. Epitopes sequence is in black. The adjuvant sequence is highlighted in green color, EAAAK linker sequence is highlighted in purple, AAY linkers are highlighted with blue, and GPGPG linkers are highlighted with pink; (**B**) MEV construct refined 3D structure (alpha helix: cyan color; beta strands: pink color and loops: brown color); (**C**) Ramachandran plot analysis of predicted structure where 97.7% of residues present in the most favored region.

To predict the tertiary structure of MEV, the CABS-fold server was used. The structure was refined by the GalaxyRefine2 server (Figure 5). The improved model Ramachandran plot analysis showed that 95.3% of amino acids are in the favorable region, 4.7% of the residues in the permitted region and 0.0% in the outer region. Further analyses revealed qRMSD is 0.439, poor rotamers are 0%, MolProbity is 1.416, clash score is 11.5, and Z-score is −4.74. Besides, the refined model showed 0 errors with PROCHECK validation (https://servicesn.mbi.ucla.edu/PROCHECK/). The refined model score was 82.6087 in quality check analysis through ERRAT. These results show that the refined model is of good quality.

**Figure 6:**
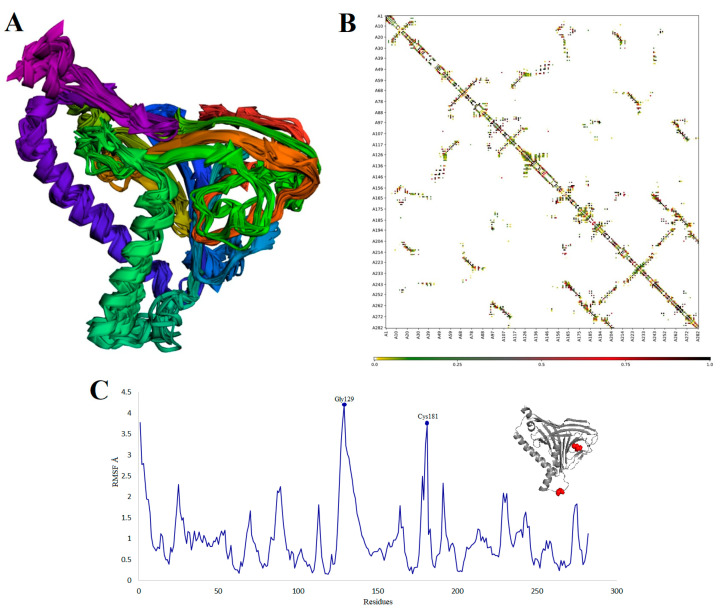
MEV structural flexibility results: (**A**) Cartoon representation of top 10 final models showing negligible fluctuation throughout; (**B**) MEV residue–residue interaction/contact map. The interactive area is represented in the central panel; (**C**) RMSF plot representing the obvious fluctuations of MEV residues during simulation. The highest fluctuations were depicted by residues Gly129 and Cys181. Both are present in loops of MEV and highlighted with red color spheres on the MEV structure (gray).

In addition, the flexibility of the MEV structure was evaluated using CABS-flex 2.0 server with 50 cycles’ simulation at 1.4 °C temperature. In between the 10 final retrieved 3D structures, regions near to N-terminal depicted lesser fluctuation compared with the regions near the C-terminal (Figure 6A). Resultant contact map presented the favorable residue interaction design for the final 10 retrieved models (Figure 6B). Finally, the root-mean-square-fluctuation (RMSF) plot disclosed the variations of all the amino acids of the MEV model from 0.0 Å to 4.5 Å (Figure 6C). The presence of fluctuations in the MEV structure indicated its high flexibility and endorses it as a potential vaccine construct. In addition, AGGRESCAN3D v.2.0 (http://biocomp.chem.uw.edu.pl/A3D2/) [92] was used to investigate the solubility and aggression propensity of MEV. It takes 3D protein structure as an input, minimizes it and calculates scores in a 10 Å radius. The results revealed that the designed MEV is highly soluble and has no potential aggression prone regions (total score: −233.8015 kcal/mol).

#### 3.2.5. B-Cells Epitopes in MEV

**Figure 7:**
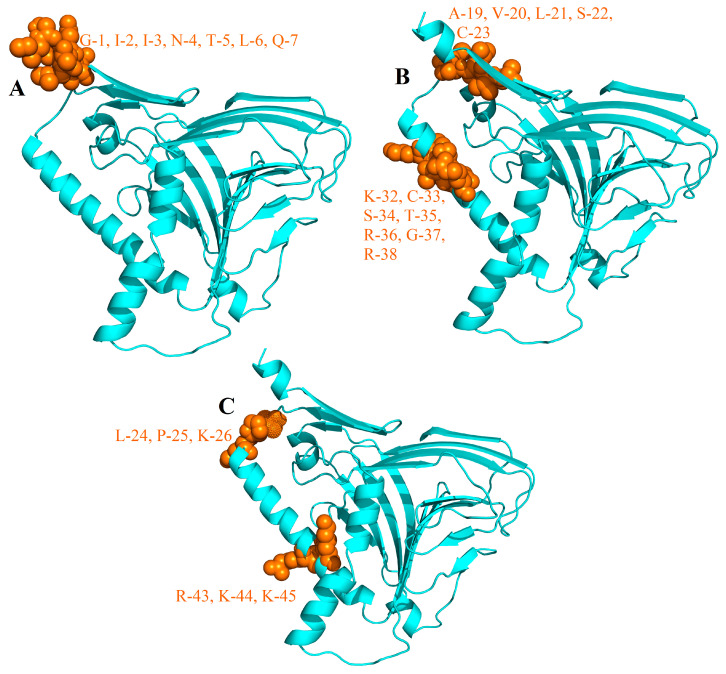
Conformational B-cell epitopes (brown) identified in the final MEV vaccine (cyan): (**A**) 7 residues (AA_1–7_); (**B**) 12 residues (AA_19–23_, AA_32–38_); (**C**) 6 residues (AA_24–26_, AA_43–45_).

B-lymphocytes besides secreting cytokines, also produce antibodies, which in return provide humoral immunity [93]. Therefore, MEV ideally should have B-cell epitopes with its domains. Three conformational/discontinuous (Figure 7) and 21 linear/continuous (Table 3) B-cell epitopes from the MEV construct sequence were predicted without altering the prediction parameters of Ellipro and ABCPred 2.0. 

#### 3.2.6. Interaction Analysis between Vaccine and TLR-3

An appropriate association between immune receptor molecules and the antigen molecule is essential to activate immune responsiveness. The HADDOCK v.2.2 server has thus been used to perform the docking of the MEV with human immune receptors TLR3. TLR3 can efficiently induce the immune response after virus recognition. The docking analysis showed the strong interactions between the MEV and TLR3. The binding score of TLR3-MEV was −151.20 kcal/mol. The statistics of docking are shown in Table 4. 

**Figure 8:**
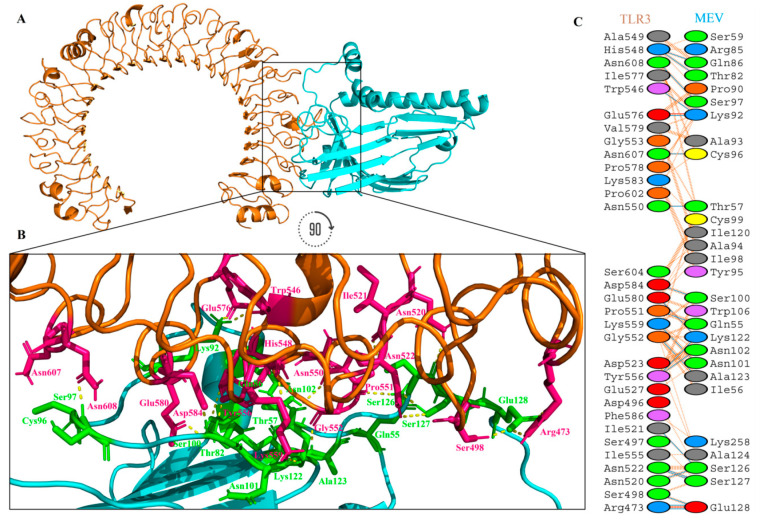
MEV construct docking with human TLR3: (**A**) TLR3-MEV docked complex in cartoon representation. TLR3 displayed with orange-brown color and MEV vaccine construct displayed with cyan color; (**B**) interacting residues illustration between MEV and TLR3 complex. Interacting residues of MEV are highlighted with green color stick representation, while interacting residues of TLR3 are highlighted with hot-pink color stick representation. Hydrogen bonds are represented with yellow color dotted lines; (**C**) All interacting residues of MEV and TLR3. Hydrogen bonds are shown with blue color lines, salt bridges are shown with red color lines and other contacts are shown with orange color lines. The colors of interacting residues are representing properties of amino acids (positive: blue, negative: red, neutral: green, aliphatic: grey, aromatic: pink, Pro&Gly: orange and Cys: yellow).

TLR3 is shown in the dark orange-brown color, while the MEV is shown in the cyan color, in Figure 8A. It was observed that MEV made 21 hydrogen bond interactions within a range of 3.00 Å with TLR3 (Figure 8B,C). MEV interacting amino acids with hydrogen bonding to TLR3 shown in green color stick representation, while similarly TLR3 amino acids interacting through hydrogen bonding with MEV shown in a hot-pink color stick representation.

#### 3.2.7. The Structural Integrity of the Vaccine-TLR3 Complex

**Figure 9:**
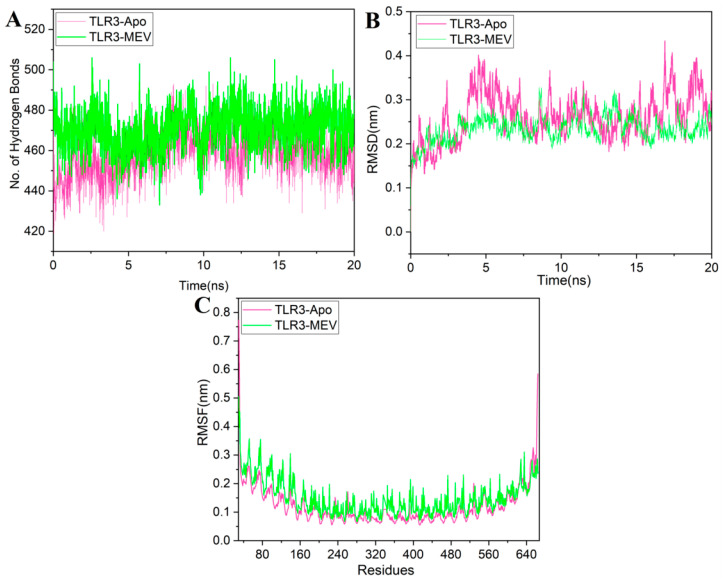
Molecular dynamics (MD) simulation at 20 ns results: (**A**) hydrogen bond interactions plot of the TLR3-Apo and TLR3-MEV complex; (**B**) the RMSD plot of the TLR3-Apo and TLR3-MEV complex; (**C**) the RMSF plot of the TLR3-Apo and TLR3-MEV complex. (TLR3-Apo RMSD represented with a pink color line while TLR3-MEV complex RMSD is represented with green color line).

MD simulation is a popular method used to analyze the micro-interactions between the ligand/vaccine and protein/receptor structures [68,94]. To further assess structural integrity, TLR3-MEV docked complex was simulated by 20 ns MD simulations followed by hydrogen bonding, root-mean-square-deviations (RMSD) and root-mean-square-fluctuations (RMSF) analyses. We also simulated TLR3 native structure without any ligands bound to it (TLR3-apo) as a control. Hydrogen bonds are the main stabilizing force for the protein and are essential to the integrity of the structure. The TLR3-MEV complex hydrogen bond interaction mode lasts stable in the overall simulation process, recommending that the proteins internal hydrogen bonds were stable throughout the 20 ns simulation (Figure 9A). To observe the structural stability of the TLR3-MEV complex, RMSD values of backbone atoms were calculated (Figure 9B). The RMSD average values for both TLR3-apo and TLR3-MEV complexes are 0.26 nm and 0.23 nm, respectively. RMSD results clearly indicated that the TLR3-MEV complex system remained more stable as compared to TLR3-apo throughout the 20 ns simulations. To further calculations, the residual and side-chain flexibility, RMSF over 20 ns time was calculated. Figure 9C depicts that there were no obvious fluctuations in TLR3-MEV complex except few fluctuations at the N-terminal and C-terminal up to RMSF 0.35 nm, while form residue 240–480 complex remained highly stable with fluctuations up to 0.15 nm. Overall, the TLR3-MEV complex showed similar behavior of fluctuations like TLR3-apo with average fluctuation values 0.14 nm and 0.12 nm, respectively. These MD simulation results validate the docking interaction analysis and endorse that MEV can strongly bind with immune receptors to generate a significant immune response against RSV.

#### 3.2.8. MM/PBSA Binding Free Energy Calculation of the Simulated Vaccine-TLR3 Complex

To clarify the energetics of the binding of MEV and TLR3 quantitatively, we carried out the MM/PBSA calculations. The calculated *ΔGbinding* of the TLR3-MEV complex is shown in Table 5. The calculated binding free energy of the TLR3-MEV complex is −2734.944 kJ/moL. Various contributions to the *ΔGbinding* reveal that the formation of the TLR3-MEV complex is driven mainly by the electrostatic interaction energy *(ΔEelec*) and the van der Waals (*ΔEvdW*) interaction. *ΔEelec* with −3451.087 ± 187.883 kJ/mol energy has high contributions than the other energies, which is also consistent with the hydrogen bond interactions between the TLR3 and vaccine.

### 3.3. Immune Simulation for Vaccine Efficacy 

**Figure 10:**
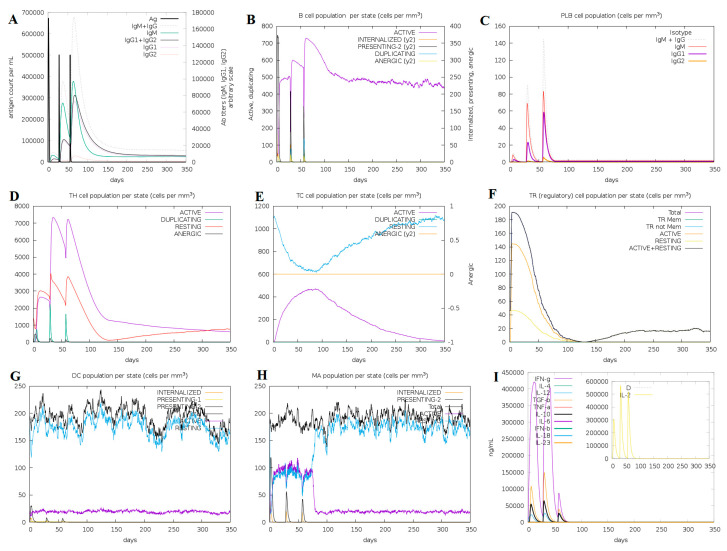
In silico immune response using MEV as antigen: (**A**) generation of immunoglobulins and B-cell isotypes upon exposure to antigen; (**B**) amount of active B-cell populations per state; (**C**) amount of plasma B-lymphocytes and their isotypes per state; (**D**) state of helper T-cell population during subsequent immune responses; (**E**) cytotoxic T-cell population per state of antigen exposure; (**F**) reduced levels of T regulatory cells; (**G**) dendritic cell population per state; (**H**) activity of macrophage population in three subsequent immune responses; (**I**) production of cytokine and interleukins in different states with the Simpson index.

The in silico mediated immune responses were adequate with real-life phenomena as shown in Figure 10. For example, both secondary and tertiary reactions were higher than the primary reaction and distinguished by the greater immunoglobulin movement i.e., (IgM, IgG1 + IgG2, and IgG + IgM antibodies) with rapid clearance of the antigen (Figure 10A). In addition, a higher activation level of B-cells, particularly Biotype IgM and IgG1, was noticed with significant memory cell development (Figure 10B,C). Likewise, the active T-cells were significantly increased in number during the secondary and tertiary reactions and gradually decreased later (Figure 10D,E). The significant levels of T regulatory cells and the continuous and rapid reproduction of macrophages and dendritic cells were noticed during the introduction of MEV. The higher levels of cytokines like IFN-γ and IL-2 were also noticed (Figure 10F,I). These observations indicated that the proposed MEV construct produced promising antiviral immune reactions.

### 3.4. In Silico Cloning within E. coli System

**Figure 11:**
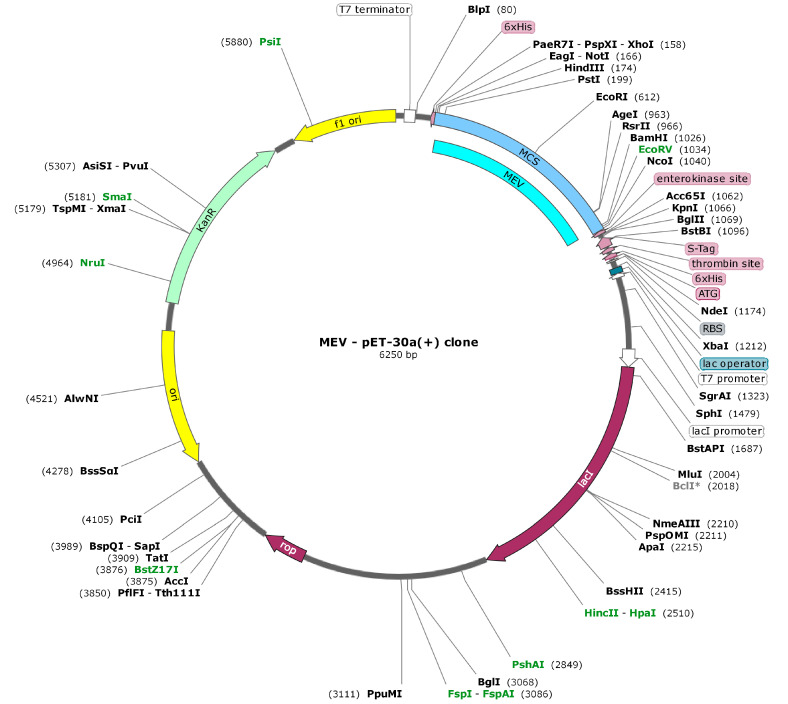
In silico cloning of codon optimized vaccine construct into *E. coli* K12 expression system. The inserted DNA sequence is shown in cyan color while keeping the plasmid back-bone in gray color.

In silico cloning was performed to ensure the expression of RSV derived MEV in commonly used *E. coli* hosts. First, codons of MEV construct were adapted as per codon utilization of *E. coli* expression system. JCAT server was used to optimize the MEV codons according to *E. coli* (strain K12). The optimized MEV construct contained 858 nucleotides (Table S7), an ideal range of GC content 51.30% (30–70%) and CAI value 1.0 (0.8–1.0) and showing the high possibility of positive protein expression and reliability. In the next step, buffer compatible restriction enzymes HindIII and BamHI restriction sites were attached to both ends of the MEV optimized nucleotide sequence to aid the cloning/purification process. Finally, the refined MEV sequence was cloned between HindIII and BamHI restriction sites at the multiple cloning-site of the pET30a (+) vector (Figure 11). The clone had a total length of 6.250 kbp.

## 4. Discussion

Vaccination is one of the most fundamental and safest ways to prevent pathogenic diseases worldwide. The present study focused on subunit vaccines as a contrast with vaccines derived through the whole pathogen. Since subunit vaccines consist of different immunogenic parts of pathogens, therefore they can produce more safe and strong immune response [17]. Vaccine development is a comparatively costly, labor-intensive, and time-consuming process. During recent years, progress in immunoinformatics has brought various servers and reliable computational tools that resulted in decreased cost and time as compared to conventional vaccine development. However, the successful formation of effective multiepitope vaccines still a great challenge because of the troubles in choice of immunodominant epitopes, suitable antigens, and an effective delivery system. Thus, for the designing of a multiepitope vaccine, the forecast of appropriate epitopes in a target antigen is highly important.

RSV is a primary source of bronchiolitis and pneumonia in babies and in the elderly, and is evaluated to cause 30 million lower respiratory tract diseases and over 60,000 deaths worldwide every year [95]. This worldwide health problem could be improved by a successful effective vaccine: however, at present, no vaccine is available against RSV [96]. A development of RSV vaccine in the 1960s, that assessed an alum-encouraged formalin-inactivated entire RSV, did not inhibit infection and caused an increase in disease seriousness upon natural RSV disease [96]. Therefore, the development of a protective and successful RSV vaccine is crucial to prevent RSV infection [97]. 

In this study, we designed an MEV, utilizing the strong B- and T-cell epitopes, obtained from the surface glycoproteins of RSV. The selected RSV proteins (F and G) had the highest antigenicity. Surface glycoproteins of RSV command the early phases of infection. G focuses on the ciliated cells of airways, and F makes the virion layer to join with a target cell membrane [98]. There is a high conservation level of F protein among all strains of RSV, giving the possibility that the F protein vaccine would secure against the range of RSV strains [98]. The RSV G protein is the additional main neutralizing-antibody focus on the outside of the RSV virion, and its expression from a Sendai or vaccinia virus vector induced a defensive immune reaction in animals [98,99]. Both G and F proteins have been evaluated as a potential vaccine candidate in several studies, and some studies reported their significant contributions in the development of subunit vaccines that can overcome challenges associated with the RSV vaccine in development process [13,23,24,25,26,27]. Therefore, we believe that the selected surface glycoproteins proteins are appropriate candidates for vaccine design against RSV, and developed MEV from these target proteins will be effective against all strains of RSV including highly prevalent RSV-A ON1 and RSV-B BA1–BA10 strains.

A perfect multiepitope vaccine would include epitopes of both B- and T-cells for encouraging a complete system of immune reactions. Thus, both kinds of epitopes were forecasted from RSV proteins and overlapping epitopes were further chosen depending on their immunogenic characteristics (i.e., antigenicity, allergenicity and toxicity). Many HLA binding-alleles were also considered for comprehensive performance. B-cells are key components of the adaptive immune system which produce antibodies against invading pathogens. Antibodies are becoming increasingly popular and useful therapeutic agent to treat infectious diseases [100,101]. However, development of therapeutic antibodies using conventional methods are labor-intensive and time-consuming, and often meet challenges due to difficulty in finding strong immunogenic epitopes [100,102]. The present study could also facilitate the researchers to computationally design therapeutic antibodies against RSV using predicted epitopes in present study [102,103]. This will help researchers to rationally design [104] functional therapeutic antibodies, instead of depending on extensive functional screening from large libraries. 

Consequently, a vaccine construct was created utilizing the strongest epitopes with suitable linkers and adjuvant. The adjuvant β-defensin is a thoroughly basic 45 amino acids long peptide that behaves both as an antimicrobial-agent and as an immunomodulator [53]. The adjuvant was connected with an EAAAK linker which is an exact α-helical linker that enhanced the bifunctional catalytic activity and firmness [51]. Further, CTL-epitopes were connected with AAY linkers and HTL epitopes with GPGPG which permit proficient dissociation and identification of each epitope [51,105]. Furthermore, the vaccine’s efficacy relies upon the populace wherein the vaccination is utilized. Particularly, the construct demonstrated ~70% coverage of the world’s population and over 90% in pathogen-attacked regions, especially in Spain, Croatia, Denmark, and Germany [106]. 

The designed vaccine construct was profoundly immunogenic, antigenic, non-toxic, and non-allergic which shows its efficacy in inducing vigorous immune reactions without causing unwanted responses. The designed vaccine contains 282 amino acid residues (30.57 kDa), where the adjuvant alone has a length of 45 residues. Interestingly, the MEV has three conformational and 21 linear B-cell epitopes overall in its domains. The final vaccine is moderately basic and can give a stable connection in the physiological pH range. The mean half-life of the construct was calculated as 30 h in vitro, >20 h in vivo and >0 h in yeast, which is consistent with already reported subunit vaccine studies [22,51,52,82,83,107]. Furthermore, results disclosed the vaccine as thermostable while the negative GRAVY recommended its hydrophilicity, proposing strong connections towards water molecules. In addition, the recombinant-proteins should be soluble on over-expression for further studies. Particularly, the designed vaccine can be easily purified because it was seen as profoundly soluble.

To effectively transport the candidate antigenic protein within the body, it should have a stable connection with immune receptors such as TLR3 [45]. In the current study, stable interactions were observed in molecular docking analysis between the vaccine and TLR3, and less energy was required for proficient binding. Moreover, during MD simulation, the vaccine-TLR3 complex was seen to be stable, where the protein-backbone experienced some microscale changes and slight variations in the adaptable areas. These outcomes were consistent with earlier studies where vaccine–receptor complex stabilization was accomplished within a similar time-scale [45,105]. The structural compactness was also assisted by hydrogen bond analysis and MM/PBSA binding free energy calculation. Therefore, our results recommended that the MEV can strongly bind with receptors.

Hypothetically, a multiepitope vaccine having B- and T-cell epitopes should manage to activate both humoral and cellular immune reactions [108]. Our vaccine demonstrated high macrophage activity and durable Th1-mediated immune responses essential to RSV in immune simulation. Furthermore, after the primary reaction, the active helper T-cell population was increasingly higher. In the current study, the highest production of IFN-γ with important IL-2 and IL-10 activities was observed. Antibodies additionally give assurance against extracellular RSV. Furthermore, we noticed an excess of active immunoglobulins, i.e., IgG, IgM, and their isotypes that may be included in isotype switching. Therefore, the imitated immune response was distinguished by higher rates of the activities of helper B-cells and T-cells. In addition, the irrelevant Simpson index (D) recommends a diverse immune reaction which is conceivable since the subunit vaccine carried various B- and T-cell epitopes [51].

The foreign gene expression may vary inside the host cell genome because of the mRNA codon inconsistency, thus, codon optimization is essential for higher expression [109]. Fortunately, both GC content and CAI value were great showing designating possible higher expression within *E. coli* K12 system. In silico restriction cloning was also performed using the pET30a (+) vector to synthesize possible candidate vaccines. For the ease of purification, that vector has both S- and His-tags as the fusion partners which are significant for easy purification [51]. Furthermore, the S-tag sequence increases the stability of proteins with their affluence of polar and charged residues [51].

This type of multiepitope-based subunit vaccines possesses remarkable qualities that give them an advantage over traditional vaccines such as: (a) it consists of B-cell, CTL and HTL epitopes, therefore it could produce both humoral and cellular immune reactions in the host; (b) it consists of epitopes focusing various HLAs, it can be identified by various T-cell receptors, thus can be effectual in a large population; (c) the risk of autoimmunity or other adverse effects is decreased as the protein sequences/epitopes which cover by human proteins and other undesirable proteins are eliminated; (d) different proteins may be targeted by a single vaccine because it consists of immunogenic areas of different proteins fused as a single peptide fragment, thus enhancing their efficacy; (e) these vaccines may provide durable immunity in hosts as they are additionally linked to an adjuvant; and (f) if these vaccines administrated through oral, intranasal or sublingual route, they can stimulate the mucosal immune response that result in stopping the pathogens before they get enter into the host body by producing defensive both B- and T-cells in mucosal and systematic environments [110,111,112,113,114,115]. Such a multiepitope vaccine may thus become an important tool in the future to fight against viral and other pathogenic infections [108]. Several research groups reported multiepitope vaccines using immunoinformatics approaches against different infectious pathogens, such as: SARS-CoV-2 [45], MERS-CoV [21], Chikungunya virus [20], Ebola virus [116], Zika virus [117], HCV [107], Flavivirus [118], Cytomegalovirus [80], HIV [109], BK virus [119], and Norovirus [120] with promising results. Since the proposed vaccine in the present study contains CTL, HTL, and B-cell epitopes, together with an adjuvant, it can stimulate both the innate and adaptive immune system of the host, which makes it a strong candidate for RSV vaccine production.

## 5. Conclusions

RSV infection has been a mysterious issue for a decade and is now considered a worldwide health problem. As such, there is no effective vaccine available for the treatment of RSV infections or any eternal cure yet. Many antiviral medications have been studied but none have clearly demonstrated effective results against the infection. Reverse vaccinology and computational techniques have been used to build a multiepitope-based subunit vaccine that could activate humoral and cellular immune responses. The proposed MEV model, coupled with computational analysis and immuno-information data, could lead to the development of a potential vaccine against RSV infection. We assume our predicted vaccine model will exert a positive effect on curing RSV infection research. However, the current study is the result of an integrated vaccinomics approach. Therefore, further lab experiments are necessary to demonstrate the efficacy and safety of the designed MEV.

## Figures and Tables

**Table 1:** Structural details of the respiratory syncytial virus (RSV) proteins predicted models.

Proteins	Tool Utilized	Template	Ramachandran Plot		
			Favored Region	Allowed Region	Disallowed Region
G	RaptorX	6blhG	89.2%	8.1%	2.7%
F	RaptorX	4mmrB	96.0%	2.9%	1.2%

**Table 2:** Final selected epitopes from RSV antigenic proteins used to design the multiepitope-based subunit vaccine (MEV) construct and their binding details with their respective human leukocytes antigen (HLA) alleles. (#: number of H-bonds)

Sr. No	Epitope	Protein	Position	HLA Alleles	Antigenicity Score	Binding Score (kcal/mol)	# H-bonds
**MHC I**							
1	KSIAQITLSILA	G	36–47	A*3201	0.6	−13.36	6
2	YLTQNPQLGISF	G	90–101	B*1502	1.3	−14.24	2
3	TTKQRQNKPPNK	G	147–158	A*3001	0.7	−21.38	12
4	CSICSNNPTCWA	G	173–184	B*5801	0.6	−14.44	4
5	CSNNPTCWAICK	G	176–186	A*1101	0.6	−19.11	12
6	SSEGNISPSQVY	G	269–280	B*4403	0.7	−14.98	6
7	ETVIEFQQKNNR	F	218–229	A*3303	1.3	−15.95	17
8	DTPCWKLHTSPL	F	310–321	B*0702	0.6	−12.49	4
9	SVSFFPLAETCK	F	348–359	A*0301	0.7	−14.85	10
10	FFPLAETCKVQS	F	351–362	B*3503	0.6	−12.93	1
11	SLYVKGEPIINF	F	466–477	B*1502	0.7	−13.82	3
12	CKARSTPVTLSK	F	550–561	A*3001	1.2	−18.88	17
13	RSTPVTLSKDQL	F	553–564	B*5801	0.7	−11.62	5
**MHC II**							
14	AIIFIASANNKVTLT	G	58–72	DRB1_0410	0.76	−12.04	5
15	IIFIASANNKVTLTT	G	59–73	DRB1_0410	0.67	−13.39	4

* is the part of alleles specific naming system, #: number of H-bonds.

**Table 3:** Linear B-cell epitopes in the final MEV vaccine construct.

B-Cell Epitope	Position	Antigenicity
LTQNPQLGISFAAY	67	1.15
SSEGNISPSQVYAA	126	0.67
IGKCSTRGRKCCRR	30	1.27
LPKEEQIGKCSTRG	24	0.75
RQNKPPNKAAYCSI	85	0.76
STPVTLSKDQLGPG	232	0.70
LAETCKAAYFFPLA	177	0.57
SPLAAYSVSFFPLA	165	0.82
NNPTCWAICKAAYS	113	0.59
YAAYETVIEFQQKN	137	1.19
LSKAAYRSTPVTLS	225	1.10
AAYCSICSNNPTCW	93	0.57
YSLYVKGEPIINFA	200	0.73
SFAAYTTKQRQNKP	76	0.85
FAAYCKARSTPVTL	212	1.22
AAYCSNNPTCWAIC	108	0.82
QKNNRAAYDTPCWK	148	0.77
VSFFPLAETCKAAY	172	0.60
GEPIINFAAYCKAR	206	0.56
AYDTPCWKLHTSPL	154	0.64
CKAAYSSEGNISPS	121	0.77

**Table 4:** Statistics of top the TLR3-MEV docked cluster.

Parameters	Value
HADDOCK v.2.2 score	−151.2 ± 2.3
Cluster size	17
RMSD from the overall lowest-energy structure	1.3 ± 0.1
Van der Waals energy	−107.5 ± 1.9
Electrostatic energy	−411.5 ± 19.7
Desolvation energy	7.0 ± 1.6
Restraints violation energy	0.0 ± 0.0
Buried Surface Area	3680.6 ± 26.9
Z-Score	0

**Table 5:** Binding energy (kcal/moL) analysis of the TLR3-MEV complex. *ΔEvdW*: van der Waals interaction energy; *ΔEelec*: electrostatic interaction energy*; ΔESA*: SAS energy; *ΔGpola*: polar solvation energy; *ΔGbinding*: binding energy.

Energy Terms	TLR3-MEV Complex
*ΔEvdW*	−394.240 ± 48.875 kJ/moL
*ΔEele*c	−3451.087 ± 187.883 kJ/moL
*ΔESA*	−59.371 ± 7.180 kJ/moL
*ΔGpolar*	1169.754 ± 248.542 kJ/moL
*ΔGbinding*	−2734.944 ± 175.446 kJ/moL

## Supplementary Materials

The following are available online at https://www.mdpi.com/2076-393X/8/2/288/s1, Figure S1: 3D structural representation of RSV antigenic proteins: (A) G protein and (B) F protein. RaptorX was used to predict these structures. These 3D structures were predicted to forecast conformational B-cell epitopes from target proteins and identifying their particular positions on proteins structures, Figure S2: 3D structures of MHC Class I and MHC Class II epitopes. These 3D structures were predicted by PEP-FOLD v.3.0 server and used as ligands in molecular docking against their respective HLA alleles, Figure S3: Secondary structure analyses of RSV MEV construct. Yellow bars are representing strands, pink bars are representing helixes and gray lines are representing coils, Table S1: RSV antigenic proteins physicochemical properties, Table S2: Prediction of RSV proteins Secondary structure through SOPMA, Table S3: Predicted CTL Epitopes. The epitopes listed in the table showed 100% conservancy among the both protein sequences included in the present study, Table S4: Predicted HTL Epitopes. The boxes colored with blue, light gray and black shows the strong, intermediate and non-binding affinities towards the respective human HLA alleles, Table S5: Predicted linear B-cell epitopes of RSV proteins, Table S6: Predicted conformational B-cell epitopes of RSV proteins, Table S7: Codon optimized nucleotide sequence of MEV construct for cloning in *E. coli* strain K12. Red color bold sequence at 5’ site (N-terminal) is representing HindIII restriction enzyme site, while green color bold sequence at 3’ site (C-terminal) is representing BamHI restriction enzyme site.
