# Supplementary material for: Multiepitope-Based Subunit Vaccine Design and Evaluation against Respiratory Syncytial Virus Using Reverse Vaccinology Approach
Source: Vaccines (Basel). 2020 Jun 8;8(2):288. doi: 10.3390/vaccines8020288 (PMC7350008; doi:10.3390/vaccines8020288)
Supplement: Supplementary file 1 [file vaccines-08-00288-s001.zip › vaccines-793521-xml-sup.docx]

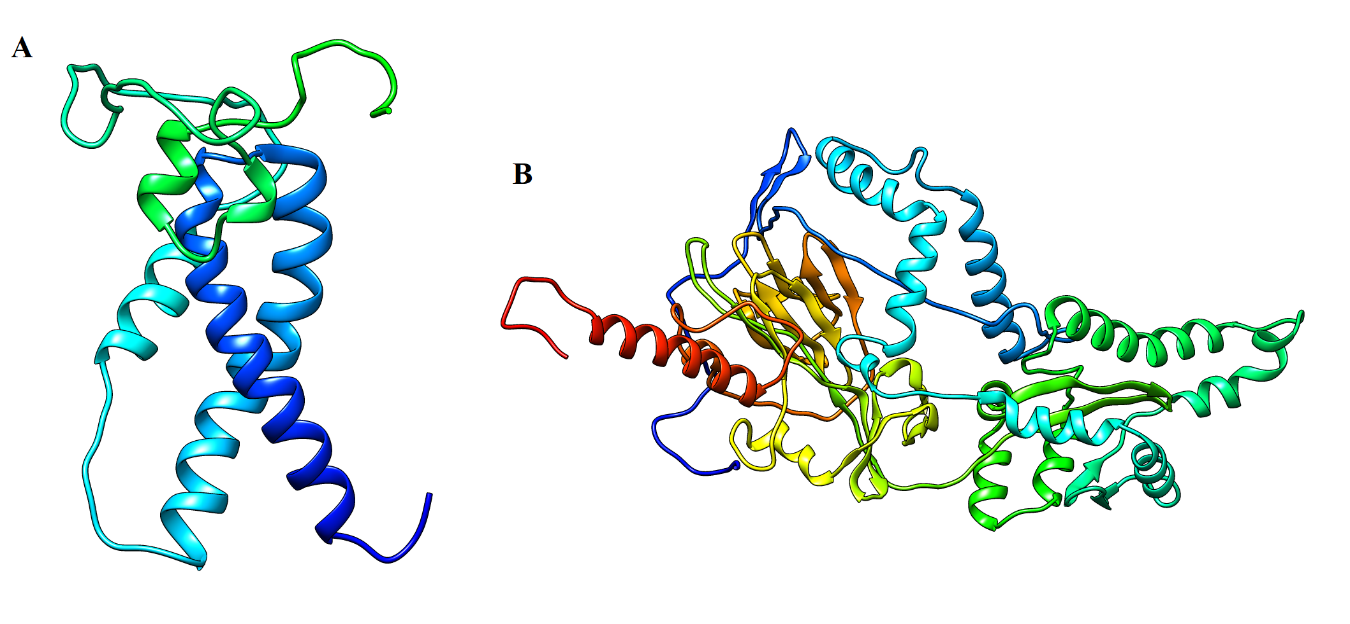


**Figure 1.** 3D structural representation of RSV antigenic proteins: (**A**) G protein and (**B**) F protein. RaptorX was used to predict these structures. These 3D structures were predicted to forecast conformational B-cell epitopes from target proteins and identifying their particular positions on proteins structures.


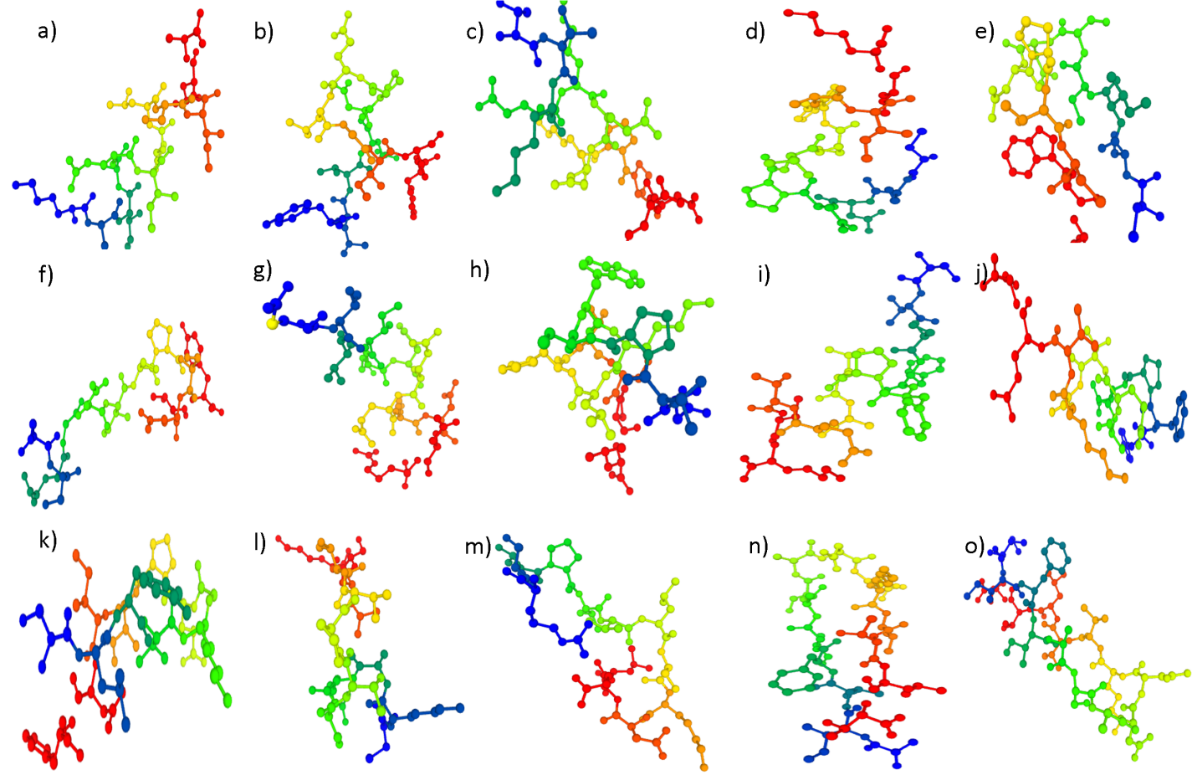


**Figure 2.** 3D structures of MHC Class I and MHC Class II epitopes. These 3D structures were predicted by PEP-FOLD v3.0 server and used as ligands in molecular docking against their respective HLA alleles. .


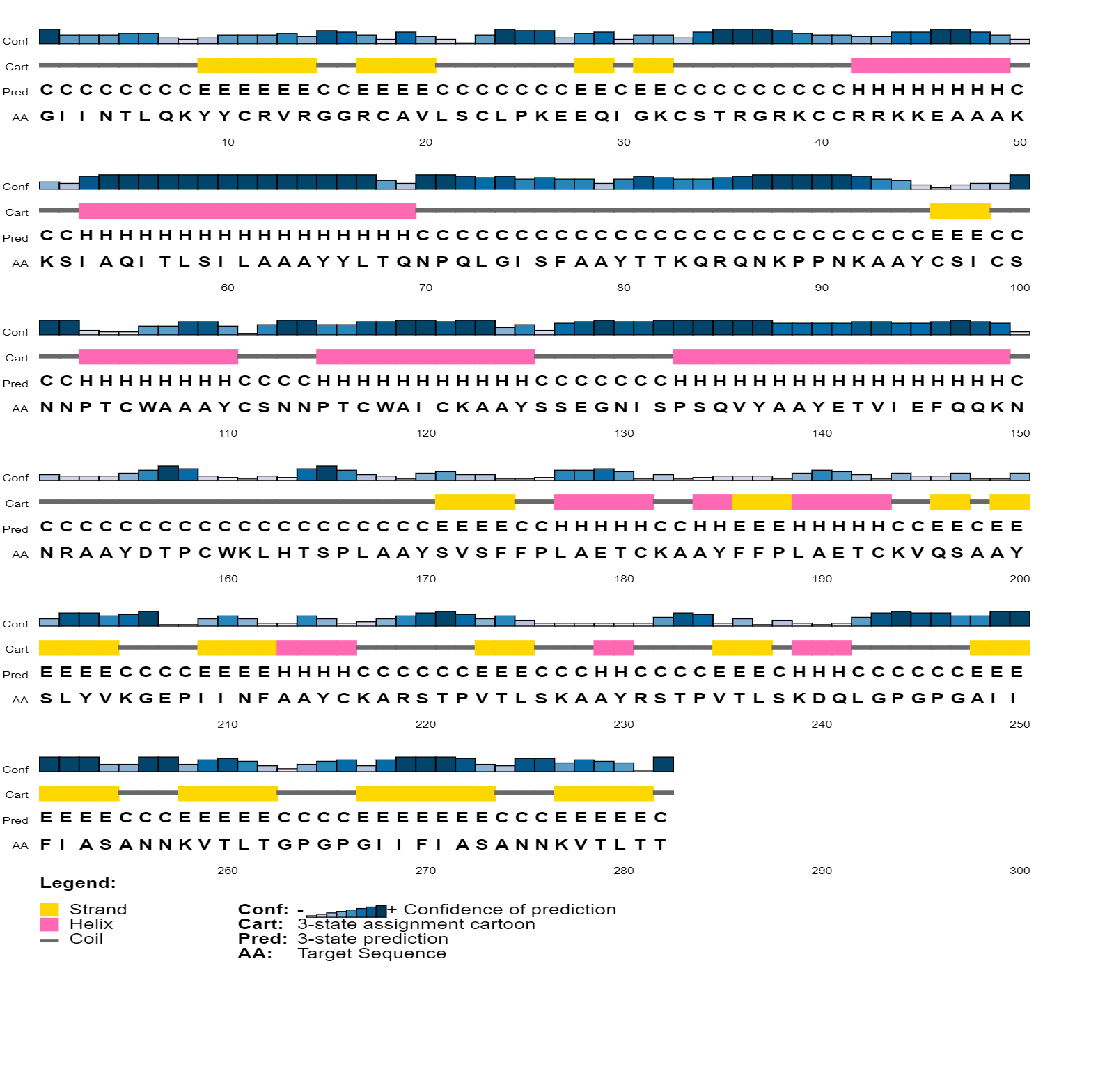


**Figure 3.** Secondary structure analyses of RSV MEV construct. Yellow bars are representing strands, pink bars are representing helixes and grey lines are representing coils.

**Table 1.** RSV antigenic proteins physiochemical properties.

| **Proteins** | **Molecular Weight** | **Theoretical pI** | **Instability index** | **Half‐Life** | **Stability Profiling** | **Aliphatic Index** | **Grand Average of Hydropathy** |
| --- | --- | --- | --- | --- | --- | --- | --- |
|  |  |  |  |  |  |  |  |
| Glycoprotein | 32767.53 | 9.85 | 37.25 | 30 hours (mammalian reticulocytes,in vitro). >20 hours (yeast, in vivo).  >10 hours (Escherichia coli, in vivo). | stable | 75.64 | -0.520 |
| Fusion protein | 63334.16 | 9.01 | 40.42 | 30 hours (mammalian reticulocytes, in vitro).  >20 hours (yeast, in vivo).  >10 hours (Escherichia coli, in vivo). | unstable | 101.50 | -0.007 |

**Table 2.** Prediction of RSV proteins Secondary structure through SOPMA.

| **Proteins** | **Sequence Length** | **α-helix** | **β-Turn** | **Random Coils** |
| --- | --- | --- | --- | --- |
| Glycoprotein | 298 | 19.80% | 3.36% | 52.35% |
| Fusion protein | 574 | 36.76% | 8.71% | 31.18% |

**Table 3.** Predicted CTL Epitopes. The epitopes listed in the table showed 100% conservancy among the both protein sequences included in the present study.

| Proteins | Peptide | Position | Allele | Antigenicity |
| --- | --- | --- | --- | --- |
| Glycoprotein | HFEVFNFVPCSI | 164-175 | HLA-B*40:01 | 0.7 |
|  | NLKSIAQITLSI | 34-45 | HLA-A*32:01 | 1.0 |
|  | KSIAQITLSILA | 36-47 | HLA-A*32:01 | 0.6 |
|  | GNPEHTSQEETL | 54-65 | HLA-B*38:01 | 0.6 |
|  | NPEHTSQEETLH | 55-66 | HLA-B*38:01 | 0.7 |
|  | LKSIAQITLSIL | 35-46 | HLA-A*32:01 | 0.6 |
|  | SSEGNISPSQVY | 269-280 | HLA-B*44:03  HLA-A*01:01  HLA-B*44:02  HLA-B*15:25  HLA-A*30:02  HLA-B*58:02  HLA-B*15:01 | 0.7 |
|  | ATSQIKNTTPTY | 79-90 | HLA-A*30:02  HLA-B*15:25  HLA-B*15:01  HLA-B*58:02  HLA-A*01:01 | 0.8 |
|  | ISPSQVYTTSEY | 274-285 | HLA-B*15:01  HLA-A*30:02  HLA-B*35:01  HLA-A*01:01  HLA-B*15:25 | 0.6 |
|  | CSICSNNPTCWA | 173-184 | HLA-B*58:01 | 0.6 |
|  | AAIIFIASANNK | 57-68 | HLA-A*11:01 | 0.7 |
|  | NNPTCWAICKRI | 178-189 | HLA-B*51:01 | 0.9 |
|  | TTKQRQNKPPNK | 147-158 | HLA-A*30:01 | 0.7 |
|  | NNDFHFEVFNFV | 160-171 | HLA-B*38:01 | 1.14 |
|  | CSNNPTCWAICK | 176-186 | HLA-A*11:01 | 0.6 |
|  | YLTQNPQLGISF | 90-101 | HLA-B*15:02 | 1.31 |
|  | LPIVNKQSCSIS | 204-215 | HLA-B*51:01 | 1.05 |
|  | DTVSVGNTLYYV | 448-459 | HLA-A*26:01  HLA-A*68:02, HLA-A*29:02,  HLA-A*25:01  HLA-A*01:01 | 0.73 |
|  | VDTVSVGNTLYY | 447-458 | HLA-A*26:01  HLA-A*01:01  HLA-A*29:02, HLA-A*25:01 | 0.6 |
| Fusion protein | LLSLIAVGLLLY | 538-549 | HLA-A*01:01  HLA-A*29:02, HLA-A*30:02 | 0.7 |
|  | SLIAVGLLLYCK | 540-551 | HLA-A*11:01, HLA-A*03:01, HLA-A*29:02, HLA-A*03:01 | 0.8 |
|  | YQSTCSAVSKGY | 33-44 | HLA-B*15:01, HLA-B*15:02 | 1.03 |
|  | KGYLSALRTGWY | 42-53 | HLA-A*01:01, HLA-A*29:02, HLA-B*58:01, HLA-B*57:01, HLA-A*30:02 | 0.5 |
|  | FFPLAETCKVQS | 351-362 | HLA-B*51:01, HLA-B*35:03 | 0.6 |
|  | GVDTVSVGNTLY | 446-457 | HLA-A*01:01, HLA-A*26:01, HLA-A*29:02 | 0.7 |
|  | YVKGEPIINFYD | 468-479 | HLA-A*25:01, HLA-A*26:01 | 0.7 |
|  | CKARSTPVTLSK | 550-561 | HLA-A*30:01  HLA-A*11:01 | 1.2 |
|  | DTPCWKLHTSPL | 310-321 | HLA-B*08:01, HLA-B*07:02 | 0.6 |
|  | RQQSYSIMSIIK | 282-293 | HLA-B*48:01, HLA-A*11:01 | 0.8 |
|  | WYCDNAGSVSFF | 341-352 | HLA-A*24:02  HLA-A*23:01  HLA-B*38:01 | 0.6 |
|  | SLSNGVSVLTSK | 180-191 | HLA-A*03:01, HLA-A*74:01, HLA-A*11:01 | 0.7 |
|  | RSTPVTLSKDQL | 553-564 | HLA-B*58:01, HLA-B*58:02 | 0.7 |
|  | LLLYCKARSTPV | 546-557 | HLA-B*08:01, HLA-A*02:01 | 1.09 |
|  | LYVKGEPIINFY | 467-478 | HLA-A*01:01  HLA-A*29:02  HLA-A*23:01,  HLA-A*26:01  HLA-A*25:01 | 0.6 |
|  | NTKEGSNICLTR | 325-336 | HLA-A*68:01  HLA-A*33:03 | 1.0 |
|  | SLYVKGEPIINF | 466-477 | HLA-A*23:01  HLA-B*15:02 | 0.7 |
|  | LRTGWYTSVITI | 48-59 | HLA-A*32:01,  HLA-B*27:02,  HLA-B*27:05 | 0.6 |
|  | SVSFFPLAETCK | 348-359 | HLA-A*03:01, HLA-A*11:01 | 0.7 |
|  | LSLIAVGLLLYC | 539-550 | HLA-A*29:02, HLA-A*01:01 | 0.8 |
|  | TELQLLMQSTPA | 91-102 | HLA-B*18:01, HLA-B*40:02 | 0.7 |
|  | ETVIEFQQKNNR | 218-229 | HLA-A*68:01, HLA-A*33:03 | 1.3 |
|  | TPCWKLHTSPLC | 311-322 | HLA-B*08:01  HLA-B*35:03, HLA-B*07:02 | 0.9 |
|  | NDMPITNDQKKL | 262-273 | HLA-B*35:03, HLA-B*37:01 | 0.6 |

**Table 4.** Predicted HTL Epitopes. The boxes colored with blue, light grey and black shows the strong, intermediate and non-binding affinities towards the respective human HLA alleles.

| **Protein** | **Peptide Sequence(position)** | **HLA DRB-1 Alleles** | | | | | | | | | | | | | | | | | | | | | | | |  | **Antigenicity** | |
| --- | --- | --- | --- | --- | --- | --- | --- | --- | --- | --- | --- | --- | --- | --- | --- | --- | --- | --- | --- | --- | --- | --- | --- | --- | --- | --- | --- | --- |
|  |  | **DRB1_0402** | **DRB1_0403** | **DRB1_0404** | **DRB1_0406** | **DRB1_0410** | **DRB1_0411** | **DRB1_0412** | **DRB1_0418** | **DRB1_0423** | **DRB1_0437** | **DRB1_0439** | **DRB1_0440** | **DRB1_0441** | **DRB1_0442** | **DRB1_0444** | | **DRB1_0449** | **DRB1_0450** | **DRB1_0453** | **DRB1_0455** | **DRB1_0456** | **DRB1_0459** | **DRB1_0467** | **DRB1_0468** | **DRB1_0470** |  |  |
| Glycoprotein | IAAIIFIASANNKVT |  |  |  |  |  |  |  |  |  |  |  |  |  |  |  | |  |  |  |  |  |  |  |  |  | 0.5 | |
|  | AAIIFIASANNKVTL |  |  |  |  |  |  |  |  |  |  |  |  |  |  |  | |  |  |  |  |  |  |  |  |  | 0.62 | |
|  | AIIFIASANNKVTLT |  |  |  |  |  |  |  |  |  |  |  |  |  |  |  | |  |  |  |  |  |  |  |  |  | 0.76 | |
|  | IIFIASANNKVTLTT |  |  |  |  |  |  |  |  |  |  |  |  |  |  |  | |  |  |  |  |  |  |  |  |  | 0.67 | |
|  | IFIASANNKVTLTTA |  |  |  |  |  |  |  |  |  |  |  |  |  |  |  | |  |  |  |  |  |  |  |  |  | 0.55 | |
|  | SQTTAILALTTPSVE |  |  |  |  |  |  |  |  |  |  |  |  |  |  |  | |  |  |  |  |  |  |  |  |  | 0.51 | |
|  | QTTAILALTTPSVES |  |  |  |  |  |  |  |  |  |  |  |  |  |  |  | |  |  |  |  |  |  |  |  |  | 0.56 | |
| Fusion Protein | VTELQLLMQSTPATN |  |  |  |  |  |  |  |  |  |  |  |  |  |  |  | |  |  |  |  |  |  |  |  |  | 0.62 | |
|  | TELQLLMQSTPATNN |  |  |  |  |  |  |  |  |  |  |  |  |  |  |  | |  |  |  |  |  |  |  |  |  | 0.64 | |
|  | ELQLLMQSTPATNNR |  |  |  |  |  |  |  |  |  |  |  |  |  |  |  | |  |  |  |  |  |  |  |  |  | 0.88 | |
|  | LQLLMQSTPATNNRA |  |  |  |  |  |  |  |  |  |  |  |  |  |  |  | |  |  |  |  |  |  |  |  |  | 0.70 | |
|  | QLLMQSTPATNNRAR |  |  |  |  |  |  |  |  |  |  |  |  |  |  |  | |  |  |  |  |  |  |  |  |  | 0.54 | |
|  | TNSELLSLINDMPIT |  |  |  |  |  |  |  |  |  |  |  |  |  |  | |  |  |  |  |  |  |  |  |  |  | 0.54 | |

**Table 5.** Predicted linear B-cell epitopes of RSV proteins.

| Proteins | B Cell Epitope (Position) | Antigenicity |
| --- | --- | --- |
| Glycoprotein | TQIQPSKPTTKQRQNK 139 | 0.68 |
|  | TSQIKNTTPTYLTQNP 80 | 0.75 |
|  | KPTIKTTKKDLKPQTT 205 | 1.19 |
|  | CSICSNNPTCWAICKR 173 | 0.83 |
|  | TKDQRTAKTLEKTWDT 4 | 0.56 |
|  | PNIRTTLLTNSTTGNP 241 | 0.67 |
|  | ESILQSTTVKTKNTTT 123 | 0.59 |
|  | SGTTSQTTAILALTTP 105 | 0.55 |
|  | SQEETLHSTSSEGNIS 260 | 0.53 |
|  | HSTSSEGNISPSQVYT 266 | 0.51 |
|  | PTTKQRQNKPPNKPNN 146 | 0.79 |
|  | VKTKNTTTTQIQPSKP 131 | 0.83 |
|  | SCLYKLNLKSIAQITL 28 | 0.77 |
|  | TYLTQNPQLGISFFNL 89 | 1.17 |
|  | HLLFISSCLYKLNLKS 22 | 0.50 |
|  | LGISFFNLSGTTSQTT 97 | 1.30 |
|  | LLTNSTTGNPEHTSQE 247 | 0.69 |
|  | AIIFIASANNKVTLTT 58 | 0.63 |
|  | KPPNKPNNDFHFEVFN 154 | 0.58 |
|  | KSIAQITLSILAMIIS 36 | 0.54 |
| Fusion protein | YGVIDTPCWKLHTSPL 306 | 0.56 |
|  | NIDIFNPKYDCKIMTS 383 | 0.78 |
|  | GSNICLTRTDRGWYCD 329 | 0.83 |
|  | SCSISNIETVIEFQQK 211 | 1.03 |
|  | TSVITIELSNIKENKC 54 | 0.61 |
|  | LLEITREFSVNAGVTT 230 | 0.60 |
|  | CKARSTPVTLSKDQLS 550 | 0.83 |
|  | LIAVGLLLYCKARSTP 541 | 1.06 |
|  | SPLCTTNTKEGSNICL 319 | 1.37 |
|  | GSVSFFPLAETCKVQS 347 | 0.73 |
|  | TRTDRGWYCDNAGSVS 335 | 0.97 |
|  | TNAITAILAAVTLCFA 8 | 0.50 |
|  | TVSVGNTLYYVNKQEG 449 | 0.83 |
|  | TCSAVSKGYLSALRTG 36 | 0.71 |
|  | FIRKSDELLHNVNAGK 505 | 0.51 |
|  | NEKINQSLAFIRKSDE 496 | 0.72 |
|  | YVSNKGVDTVSVGNTL 441 | 0.73 |
|  | IETVIEFQQKNNRLLE 217 | 0.88 |
|  | SLGAIVSCYGKTKCTA 409 | 0.77 |
|  | NIKENKCNGTDAKVKL 63 | 0.52 |
|  | VKGEPIINFYDPLVFP 469 | 0.55 |
|  | QEGKSLYVKGEPIINF 462 | 0.50 |
|  | GSAIASGIAVSKVLHL 145 | 0.58 |
|  | PRFMNYTLNNTKNTNV 112 | 0.89 |
|  | GVSVLTSKVLDLKNYI 184 | 0.76 |
|  | SDEFDASISQVNEKIN 485 | 0.66 |
|  | PIVNKQSCSISNIETV 205 | 0.74 |
|  | TLYYVNKQEGKSLYVK 455 | 0.52 |
|  | NVTLSKKRKRRFLGFL 126 | 1.20 |
|  | NVNAGKSTTNIMITTI 515 | 0.73 |
|  | PVTLSKDQLSGINNIA 556 | 0.62 |

**Table 6.** Predicted conformational B-cell epitopes of RSV proteins.

| Protein | Conformational B-Cell Epitopes | 3D Structure |
| --- | --- | --- |
| Glycoprotein | C:T104, C:C105, C:W106 | 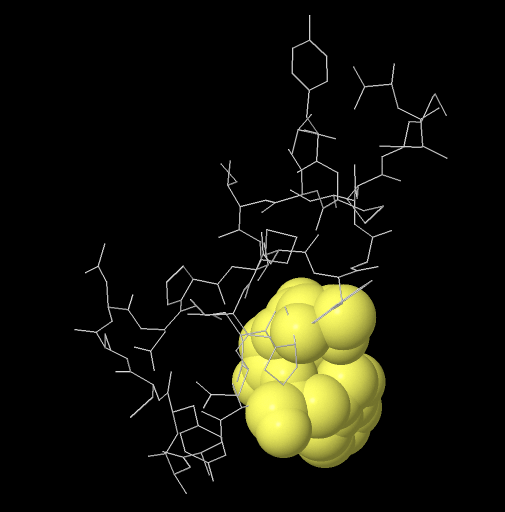 |
|  | C:A94, C:Y95, C:C121, C:K122, C:A123, C:A124 | 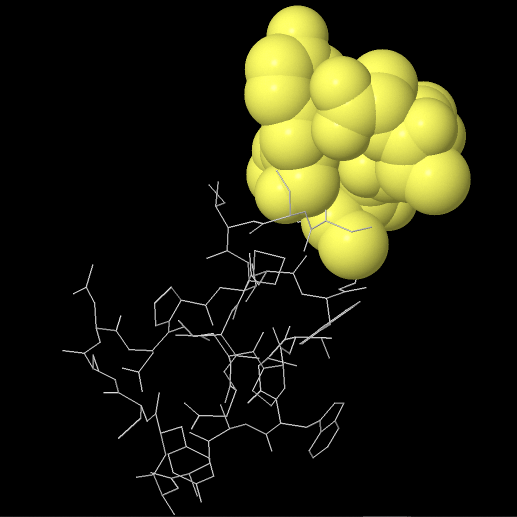 |
|  | C:N101, C:A107, C:A108, C:A109, C:Y110, C:C111, C:S112, C:N113, C:N114, C:P115 | 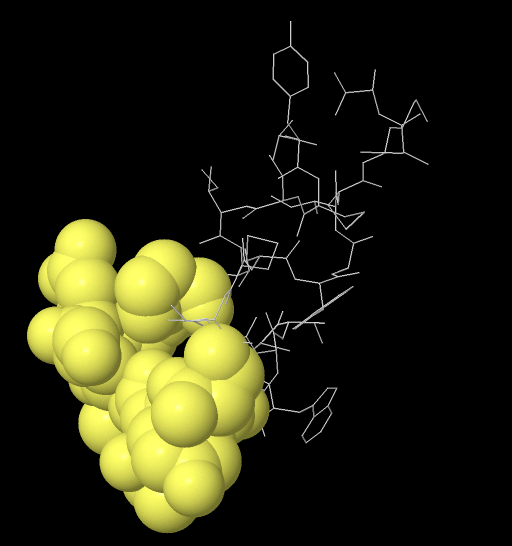 |
| Fusion Protein | :M1, _:E2, _:L3, _:P4, _:I5, _:L6, _:K7, _:T8, _:N9, _:A10, _:I11, _:T12, _:A13, _:I14, _:L15, _:A16, _:A17, _:V18, _:T19, _:L20, _:C21, _:F22, _:A23, _:S24, _:S25, _:Q26, _:N27, _:I28, _:T29, _:E30, _:E31, _:F32, _:Y33, _:Q34, _:S35, _:T36, _:C37, _:S38, _:A39, _:V40, _:S41, _:K42, _:G43, _:Y44, _:L45, _:S46, _:A47, _:L48, _:K421 | 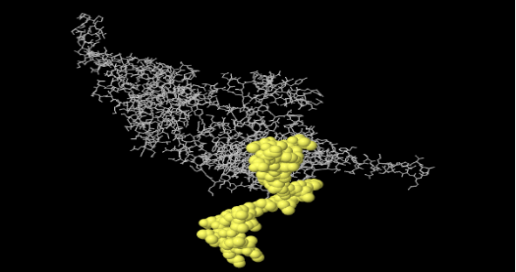 |
|  | :I332, _:D392, _:E472, _:F477, _:Y478, _:D479, _:P480, _:L481, _:V482, _:F483, _:P484, _:S485, _:S493, _:Q494, _:V495, _:N496, _:E497, _:K498, _:I499, _:N500, _:Q501, _:S502, _:L503, _:A504, _:F505, _:I506, _:R507, _:K508, _:S509, _:D510, _:L512, _:L513, _:H514, _:N515, _:V516, _:N517, _:A518, _:G519, _:K520, _:S521, _:T522, _:T523 | 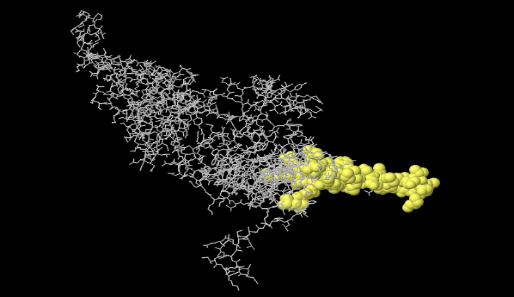 |
|  | A153, _:V154, _:S155, _:K156, _:V157, _:L158, _:H159, _:L160, _:E161, _:G162, _:E163, _:V164, _:N165, _:K166, _:I167, _:K168, _:S169, _:A170, _:L171, _:L172, _:S173, _:T174, _:N175, _:K176, _:A177, _:V178, _:V179, _:S180, _:L181, _:S182, _:N183, _:G184, _:V185, _:S186, _:V187, _:L188, _:T189, _:S190, _:K191, _:V192, _:L193, _:D194, _:L195, _:K196, _:N197, _:Y198, _:I199, _:D200, _:K201, _:Q202, _:L203, _:L204, _:P205, _:I206, _:V207, _:N208, _:K209, _:Q210, _:S211, _:C212, _:S213, _:I214, _:S215, _:N216, _:I217, _:E218, _:T219, _:V220, _:I221, _:E222, _:F223, _:Q224, _:Q225, _:K226, _:N227, _:N228, _:L230, _:L231, _:T234, _:S290, _:I291, _:I292, _:K293, _:E294, _:E295, _:V296, _:L297, _:A298 | *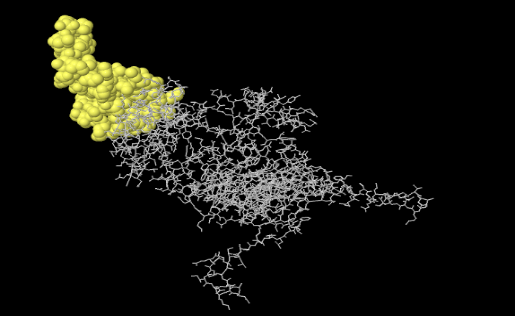* |
|  | :R49, _:T50, _:G51, _:W52, _:Q94, _:L95, _:L96, _:M97, _:Q98, _:S99, _:T100, _:P101, _:A102, _:T103, _:N104, _:N105, _:R106, _:A107, _:R108, _:R109, _:E110, _:L111, _:P112, _:R113, _:F114, _:M115, _:N116, _:Y117, _:L119, _:N120, _:N121, _:T122, _:K123, _:G418, _:K419, _:T420 | *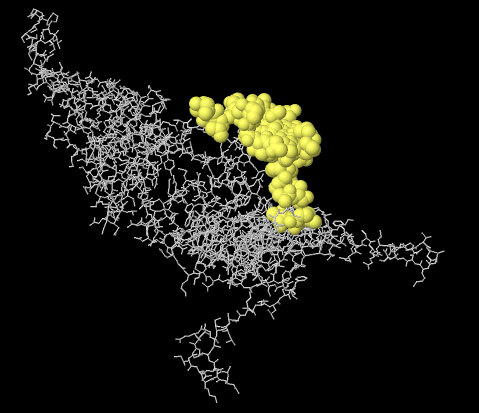* |
|  | :S259, _:N262, _:D263, _:M264, _:P265, _:I266, _:T267, _:N268, _:D269, _:K271 | *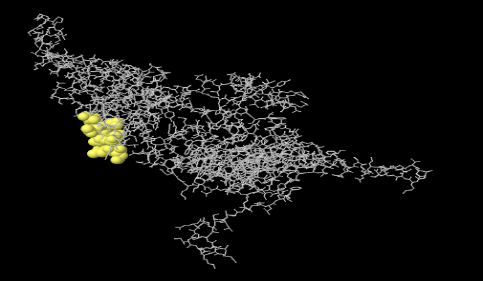* |
|  | _:N426, _:K427, _:D428, _:R429, _:G430, _:I431, _:I432, _:G446, _:V447, _:D448 | *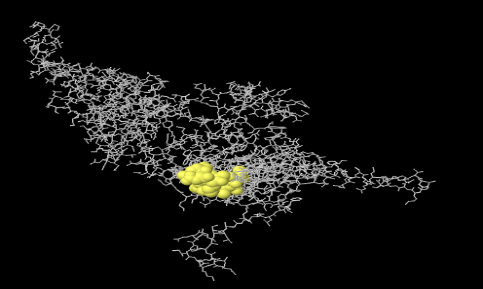* |
|  | :A346, _:G347, _:S348, _:P376, _:S377, _:E378, _:L381, _:I384, _:D385, _:I386, _:N388, _:P389, _:K390 | *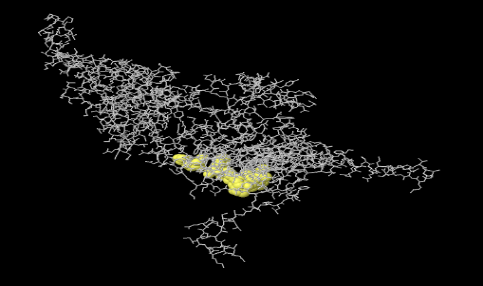* |
|  | C69, _:N70, _:G71, _:T72, _:D73, _:A74, _:K75, _:V76, _:K77 | *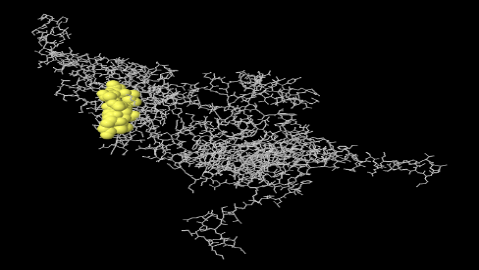* |
|  | :T323, _:T324, _:N325, _:T326, _:K327, _:E328, _:G329, _:S330, _:N331, _:K399 | *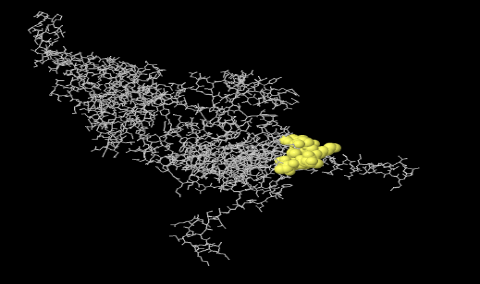* |

**Table 7.** Codon optimized nucleotide sequence of MEV construct for cloning in *E. coli* strain K12. Red color bold sequence at 5` site (N-terminal) is representing HindIII restriction enzyme site, while green color bold sequence at 3` site (C-terminal) is representing BamHI restriction enzyme site.

| **AAGCTT**GGTATCATCAACACCCTGCAGAAATACTACTGCCGTGTTCGTGGTGGTCGTTGCGCTGTTCTGTCTTGCCTGCCGAAAGAAGAACAGATCGGTAAATGCTCTACCCGTGGTCGTAAATGCTGCCGTCGTAAAAAAGAAGCTGCTGCTAAAAAATCTATCGCTCAGATCACCCTGTCTATCCTGGCTGCTGCTTACTACCTGACCCAGAACCCGCAGCTGGGTATCTCTTTCGCTGCTTACACCACCAAACAGCGTCAGAACAAACCGCCGAACAAAGCTGCTTACTGCTCTATCTGCTCTAACAACCCGACCTGCTGGGCTGCTGCTTACTGCTCTAACAACCCGACCTGCTGGGCTATCTGCAAAGCTGCTTACTCTTCTGAAGGTAACATCTCTCCGTCTCAGGTTTACGCTGCTTACGAAACCGTTATCGAATTCCAGCAGAAAAACAACCGTGCTGCTTACGACACCCCGTGCTGGAAACTGCACACCTCTCCGCTGGCTGCTTACTCTGTTTCTTTCTTCCCGCTGGCTGAAACCTGCAAAGCTGCTTACTTCTTCCCGCTGGCTGAAACCTGCAAAGTTCAGTCTGCTGCTTACTCTCTGTACGTTAAAGGTGAACCGATCATCAACTTCGCTGCTTACTGCAAAGCTCGTTCTACCCCGGTTACCCTGTCTAAAGCTGCTTACCGTTCTACCCCGGTTACCCTGTCTAAAGACCAGCTGGGTCCGGGTCCGGGTGCTATCATCTTCATCGCTTCTGCTAACAACAAAGTTACCCTGACCGGTCCGGGTCCGGGTATCATCTTCATCGCTTCTGCTAACAACAAAGTTACCCTGACCACCG**GATCC** |
| --- |

| 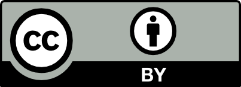 | © 2020 by the authors. Submitted for possible open access publication under the terms and conditions of the Creative Commons Attribution (CC BY) license (http://creativecommons.org/licenses/by/4.0/). |
| --- | --- |
