# Supplementary material for: Multiepitope-Based Subunit Vaccine Design and Evaluation against Respiratory Syncytial Virus Using Reverse Vaccinology Approach
Source: Vaccines (Basel). 2020 Jun 8;8(2):288. doi: 10.3390/vaccines8020288 (PMC7350008; doi:10.3390/vaccines8020288)
Supplement: Supplementary file 1 [file vaccines-08-00288-s001.zip › vaccines-793521-xml-sup.pdf]

vaccines

MDPI

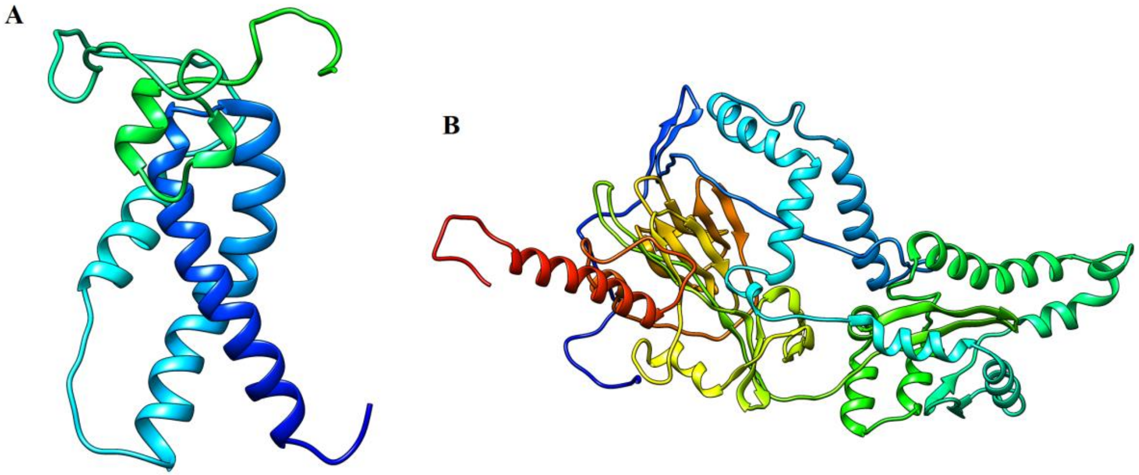

Figure 1. 3D structural representation of RSV antigenic proteins: (**A**) G protein and (**B**) F protein. RaptorX was used to predict these structures. These 3D structures were predicted to forecast conformational B-cell epitopes from target proteins and identifying their particular positions on proteins structures.

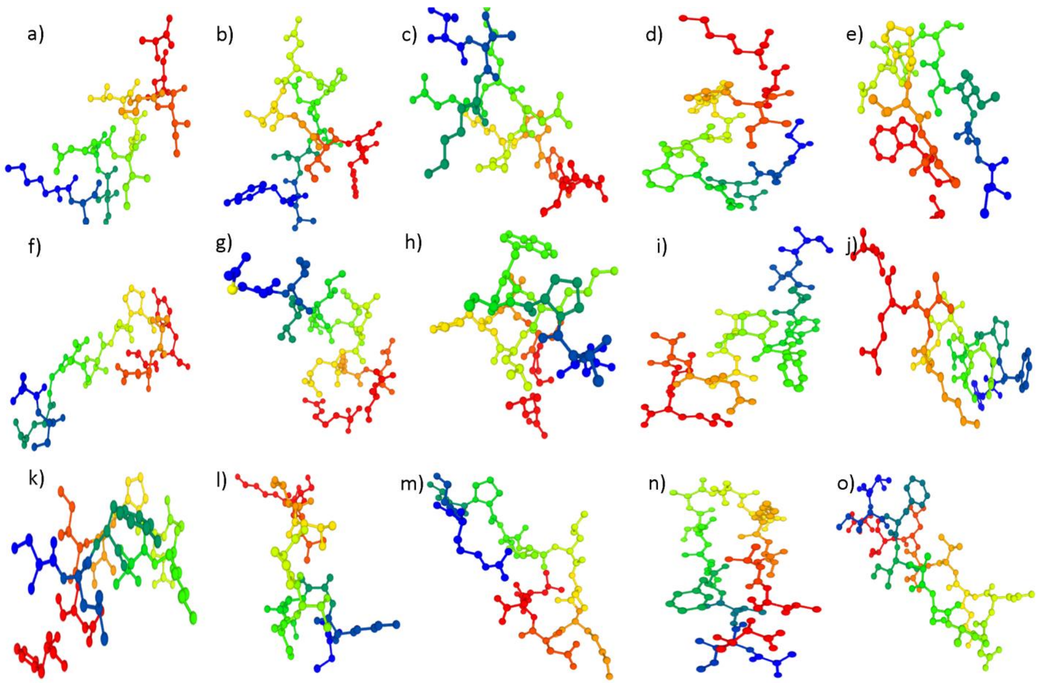

Figure 2. 3D structures of MHC Class I and MHC Class II epitopes. These 3D structures were predicted by PEP-FOLD v3.0 server and used as ligands in molecular docking against their respective HLA alleles. .

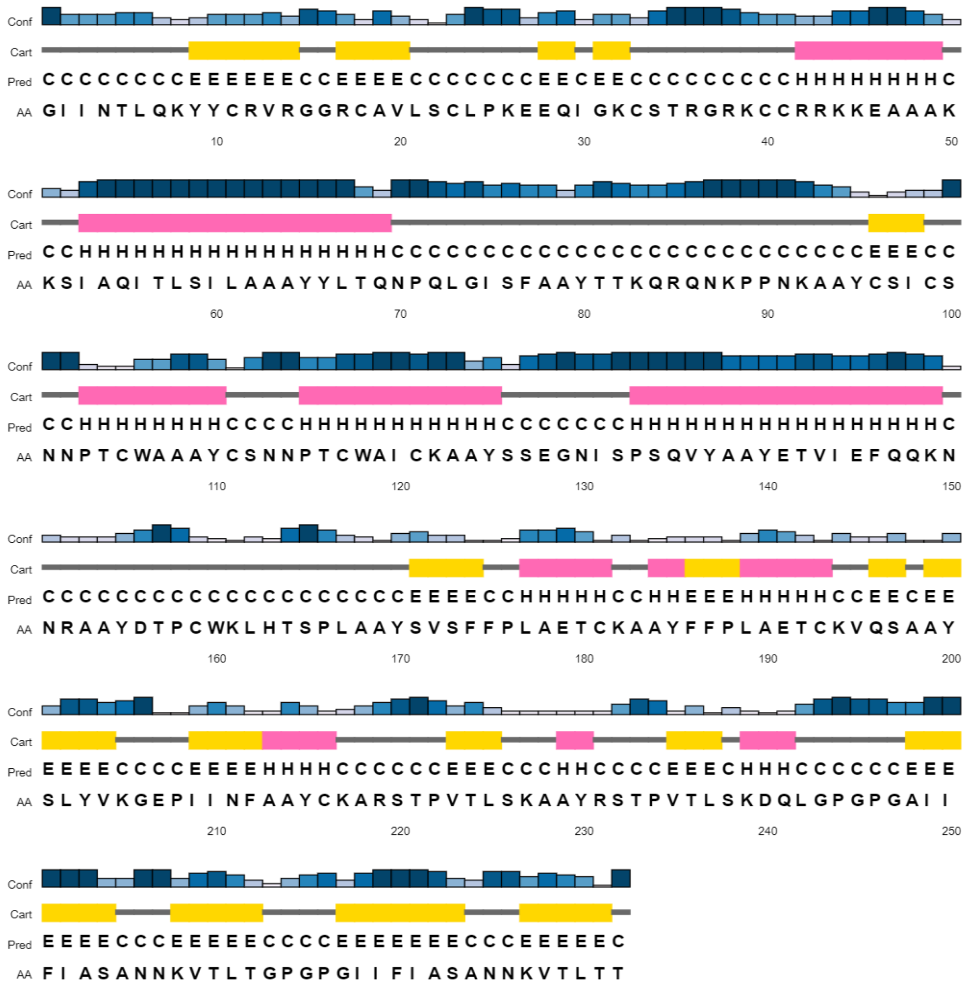

Figure 3. Secondary structure analyses of RSV MEV construct. Yellow bars are representing strands, pink bars are representing helixes and grey lines are representing coils.

Table 1. RSV antigenic proteins physiochemical properties.

Proteins	Molecular Weight	Theoretical pI	Instability index	Half-Life	Stability Profiling	Aliphatic Index	Grand Average of Hydropathy
Glycoprotein	32767.53	9.85	37.25	30 hours (mammalian reticulocytes,in vitro). >20 hours (yeast, in vivo). >10 hours (Escherichia coli, in vivo).	stable	75.64	-0.520
Fusion protein	63334.16	9.01	40.42	30 hours (mammalian reticulocytes, in vitro). >20 hours (yeast, in vivo). >10 hours (Escherichia coli, in vivo).	unstable	101.50	-0.007

Table 2. Prediction of RSV proteins Secondary structure through SOPMA.

Proteins	Sequence Length	α-helix	β-Turn	Random Coils
Glycoprotein	298	19.80%	3.36%	52.35%
Fusion protein	574	36.76%	8.71%	31.18%

Table 3. Predicted CTL Epitopes. The epitopes listed in the table showed 100% conservancy among the both protein sequences included in the present study.

Proteins	Peptide	Position	Allele	Antigenicity
**Glycoprotein**	HFEVFNFVPCSI	164-175	HLA-B*40:01	0.7
	NLKSIAQITLSI	34-45	HLA-A*32:01	1.0
	KSIAQITLSILA	36-47	HLA-A*32:01	0.6
	GNPEHTSQEETL	54-65	HLA-B*38:01	0.6
	NPEHTSQEETLH	55-66	HLA-B*38:01	0.7
	LKSIAQITLSIL	35-46	HLA-A*32:01	0.6
	SSEGNISPSQVY	269-280	HLA-B*44:03<br>HLA-A*01:01<br>HLA-B*44:02<br>HLA-B*15:25<br>HLA-A*30:02<br>HLA-B*58:02<br>HLA-B*15:01	0.7
	ATSQIKNTTPTY	79-90	HLA-A*30:02<br>HLA-B*15:25<br>HLA-B*15:01<br>HLA-B*58:02<br>HLA-A*01:01	0.8
	ISPSQVYTTSEY	274-285	HLA-B*15:01<br>HLA-A*30:02<br>HLA-B*35:01<br>HLA-A*01:01<br>HLA-B*15:25	0.6
	CSICSNNPTCWA	173-184	HLA-B*58:01	0.6
	AAIIFIASANNK	57-68	HLA-A*11:01	0.7
	NNPTCWAICKRI	178-189	HLA-B*51:01	0.9
	TTKQRQNKPPNK	147-158	HLA-A*30:01	0.7
	NNDFHFEVFNFV	160-171	HLA-B*38:01	1.14
	CSNNPTCWAICK	176-186	HLA-A*11:01	0.6
	YLTQNPQLGISF	90-101	HLA-B*15:02	1.31
	LPIVNKQSCSIS	204-215	HLA-B*51:01	1.05

	DTVSVGNTLYYV	448-459	HLA-A*26:01 HLA-A*68:02, HLA-A*29:02, HLA-A*25:01	0.73
	VDTVSVGNTLYY	447-458	HLA-A*01:01 HLA-A*26:01 HLA-A*01:01 HLA-A*29:02, HLA-A*25:01	0.6
	LLSLIAVGLLLY	538-549	HLA-A*01:01 HLA-A*29:02, HLA-A*30:02	0.7
	SLIAVGLLLYCK	540-551	HLA-A*11:01, HLA-A*03:01, HLA-A*29:02, HLA-A*03:01	0.8
	YQSTCSAVSKGY	33-44	HLA-B*15:01, HLA-B*15:02	1.03
	KGYLSALRTGWY	42-53	HLA-A*01:01, HLA-A*29:02, HLA-B*58:01, HLA-B*57:01, HLA-A*30:02	0.5
**Fusion protein**	FFPLAETCKVQS	351-362	HLA-B*51:01, HLA-B*35:03	0.6
	GVDTVSVGNTLY	446-457	HLA-A*01:01, HLA-A*26:01, HLA-A*29:02	0.7
	YVKGEPIINFYD	468-479	HLA-A*25:01, HLA-A*26:01	0.7
	CKARSTPVTLSK	550-561	HLA-A*30:01 HLA-A*11:01	1.2
	DTPCWKLHTSPL	310-321	HLA-B*08:01, HLA-B*07:02	0.6
	RQQSYSIMSIIK	282-293	HLA-B*48:01, HLA-A*11:01	0.8

WYCDNAGSVSFF	341-352	HLA-A*24:02<br>HLA-A*23:01<br>HLA-B*38:01	0.6
SLSNGVSVLTSK	180-191	HLA-A*03:01,<br>HLA-A*74:01,<br>HLA-A*11:01	0.7
RSTPVTLSKDQL	553-564	HLA-B*58:01,<br>HLA-B*58:02	0.7
LLLYCKARSTPV	546-557	HLA-B*08:01,<br>HLA-A*02:01	1.09
LYVKGEPIINFY	467-478	HLA-A*01:01<br>HLA-A*29:02<br>HLA-A*23:01,<br>HLA-A*26:01	0.6
NTKEGSNICLTR	325-336	HLA-A*25:01<br>HLA-A*68:01<br>HLA-A*33:03	1.0
SLYVKGEPIINF	466-477	HLA-A*23:01<br>HLA-B*15:02	0.7
LRTGWYTSVITI	48-59	HLA-A*32:01,<br>HLA-B*27:02,<br>HLA-B*27:05	0.6
SVSFFPLAETCK	348-359	HLA-A*03:01,<br>HLA-A*11:01	0.7
LSLIAVGLLLYC	539-550	HLA-A*29:02,<br>HLA-A*01:01	0.8
TELQLLMQSTPA	91-102	HLA-B*18:01,<br>HLA-B*40:02	0.7
ETVIEFQQKNNR	218-229	HLA-A*68:01,<br>HLA-A*33:03	1.3
TPCWKLHTSPLC	311-322	HLA-B*08:01<br>HLA-B*35:03,<br>HLA-B*07:02	0.9
NDMPITNDQKKL	262-273	HLA-B*35:03,<br>HLA-B*37:01	0.6

Table 4. Predicted HTL Epitopes. The boxes colored with blue, light grey and black shows the strong, intermediate and non-binding affinities towards the respective human HLA alleles.

Protein	Peptide Sequence(position)	HLA DRB-1 Alleles																								Antigenicity
		DRB1_0402	DRB1_0403	DRB1_0404	DRB1_0406	DRB1_0410	DRB1_0411	DRB1_0412	DRB1_0418	DRB1_0423	DRB1_0437	DRB1_0439	DRB1_0440	DRB1_0441	DRB1_0442	DRB1_0444	DRB1_0449	DRB1_0450	DRB1_0453	DRB1_0455	DRB1_0456	DRB1_0459	DRB1_0467	DRB1_0468	DRB1_0470	
Glycoprotein	IAAIIFIASANNKVT																									0.5
	AAIIFIASANNKVTL																									0.62
	AIIFIASANNKVTLT																									0.76
	IIFIASANNKVTLTT																									0.67
	IFIASANNKVTLTTA																									0.55
	SQTTAILALTTPSVE																									0.51
	QTTAILALTTPSVES																									0.56
Fusion Protein	VTELQLLMQSTPATN																									0.62
	TELQLLMQSTPATNN																									0.64
	ELQLLMQSTPATNNR																									0.88
	LQLLMQSTPATNNRA																									0.70
	QLLMQSTPATNNRAR																									0.54
	TNSELLSLINDMPIT																									0.54

Table 5. Predicted linear B-cell epitopes of RSV proteins.

Proteins	B Cell Epitope (Position)	Antigenicity
**Glycoprotein**	TQIQPSKPTTKQRQNK 139	0.68
	TSQIKNTTPTYLTQNP 80	0.75
	KPTIKTTKKDLKPQTT 205	1.19
	CSICSNNPTCWAICKR 173	0.83
	TKDQRTAKTLEKTWDT 4	0.56
	PNIRTTLLTNSTTGNP 241	0.67
	ESILQSTTVKTKNTTT 123	0.59
	SGTTSQTTAILALTTP 105	0.55
	SQEETLHSTSSEGNIS 260	0.53
	HSTSSEGNISPSQVYT 266	0.51
	PTTKQRQNKPPNKPNN 146	0.79
	VKTKNTTTTQIQPSKP 131	0.83
	SCLYKLNLKSIAQITL 28	0.77
	TYLTQNPQLGISFFNL 89	1.17
	HLLFISSCLYKLNLKS 22	0.50
	LGISFFNLSGTTSQTT 97	1.30
	LLTNSTTGNPEHTSQE 247	0.69
	AIIFIASANNKVTLTT 58	0.63
	KPPNKPNNDFHFEVFN 154	0.58
	KSIAQITLSILAMIIS 36	0.54
**Fusion protein**	YGVIDTPCWKLHTSPL 306	0.56
	NIDIFNPKYDCKIMTS 383	0.78
	GSNICLTRTDRGWYCD 329	0.83
	SCSISNIETVIEFQQK 211	1.03
	TSVITIELSNIKENKC 54	0.61
	LLEITREFSVNAGVTT 230	0.60
	CKARSTPVTLSKDQLS 550	0.83
	LIAVGLLLYCKARSTP 541	1.06
	SPLCTTNTKEGSNICL 319	1.37
	GSVSFFPLAETCKVQS 347	0.73
	TRTDRGWYCDNAGSVS 335	0.97
	TNAITAILAAVTLCFA 8	0.50
	TVSVGNTLYYVNKQEG 449	0.83

TCSAVSKGYLSALRTG 36	0.71
FIRKSDELLHNVNAGK 505	0.51
NEKINQSLAFIRKSDE 496	0.72
YVSNKGVDTVSVGNTL 441	0.73
IETVIEFQQKNNRLLE 217	0.88
SLGAIVSCYGKTKCTA 409	0.77
NIKENKCNGTDAKVKL 63	0.52
VKGEPIINFYDPLVFP 469	0.55
QEGKSLYVKGEPIINF 462	0.50
GSAIASGIAVSKVLHL 145	0.58
PRFMNYTLNNTKNTNV 112	0.89
GVSVLTSKVLDLKNYI 184	0.76
SDEFDASISQVNEKIN 485	0.66
PIVNKQSCSISNIETV 205	0.74
TLYYVNKQEGKSLYVK 455	0.52
NVTLSKKRKRRFLGFL 126	1.20
NVNAGKSTTNIMITTI 515	0.73
PVTLSKDQLSGINNIA 556	0.62

Table 6. Predicted conformational B-cell epitopes of RSV proteins.

Protein	Conformational B-Cell Epitopes	3D Structure
	C:T104, C:C105, C:W106	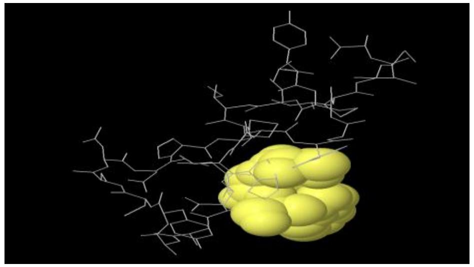
Glycoprotein	C:A94, C:Y95, C:C121, C:K122, C:A123, C:A124	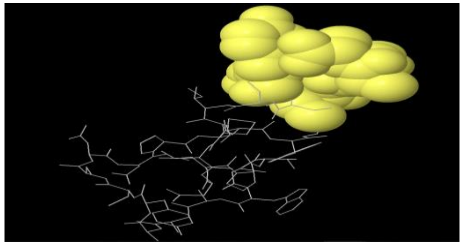
	C:N101, C:A107, C:A108, C:A109, C:Y110, C:C111, C:S112, C:N113, C:N114, C:P115	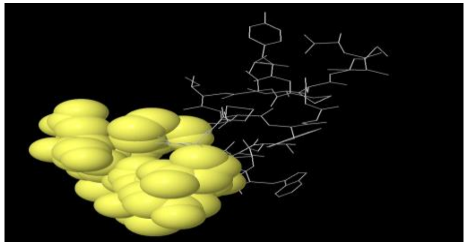

	:M1, _:E2, _:L3, _:P4, _:I5, _:L6, _:K7, _:T8, _:N9, _:A10, _:I11, _:T12, _:A13, _:I14, _:L15, _:A16, _:A17, _:V18, _:T19, _:L20, _:C21, _:F22, _:A23, _:S24, _:S25, _:Q26, _:N27, _:I28, _:T29, _:E30, _:E31, _:F32, _:Y33, _:Q34, _:S35, _:T36, _:C37, _:S38, _:A39, _:V40, _:S41, _:K42, _:G43, _:Y44, _:L45, _:S46, _:A47, _:L48, _:K421	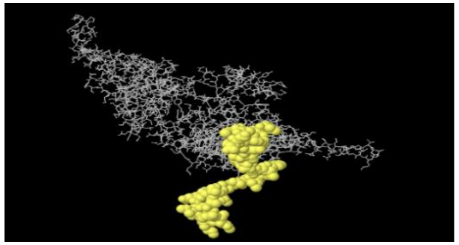
**Fusion Protein**	:I332, _:D392, _:E472, _:F477, _:Y478, _:D479, _:P480, _:L481, _:V482, _:F483, _:P484, _:S485, _:S493, _:Q494, _:V495, _:N496, _:E497, _:K498, _:I499, _:N500, _:Q501, _:S502, _:L503, _:A504, _:F505, _:I506, _:R507, _:K508, _:S509, _:D510, _:L512, _:L513, _:H514, _:N515, _:V516, _:N517, _:A518, _:G519, _:K520, _:S521, _:T522, _:T523	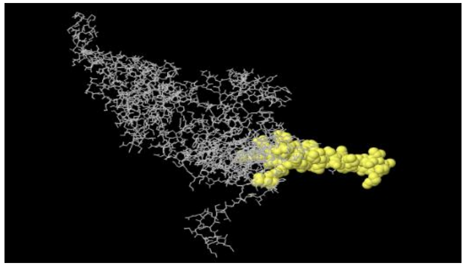
	A153, _:V154, _:S155, _:K156, _:V157, _:L158, _:H159, _:L160, _:E161, _:G162, _:E163, _:V164, _:N165, _:K166, _:I167, _:K168, _:S169, _:A170, _:L171, _:L172, _:S173, _:T174, _:N175, _:K176, _:A177, _:V178, _:V179, _:S180, _:L181, _:S182, _:N183, _:G184, _:V185, _:S186, _:V187, _:L188, _:T189, _:S190, _:K191, _:V192, _:L193, _:D194, _:L195, _:K196, _:N197, _:Y198, _:I199, _:D200, _:K201, _:Q202, _:L203, _:L204, _:P205, _:I206, _:V207, _:N208, _:K209, _:Q210, _:S211, _:C212, _:S213, _:I214, _:S215, _:N216, _:I217, _:E218, _:T219, _:V220, _:I221, _:E222, _:F223, _:Q224, _:Q225, _:K226, _:N227, _:N228, _:L230, _:L231, _:T234, _:S290, _:I291, _:I292, _:K293, _:E294, _:E295, _:V296, _:L297, _:A298	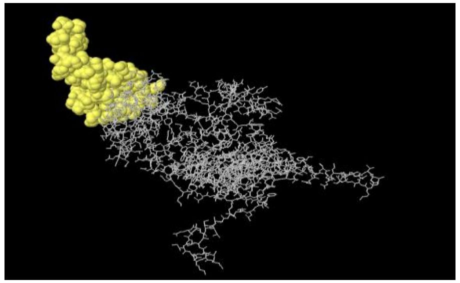

:R49, _:T50, _:G51, _:W52, _:Q94, _:L95, _:L96, _:M97, _:Q98, _:S99, _:T100, _:P101, _:A102, _:T103, _:N104, _:N105, _:R106, _:A107, _:R108, _:R109, _:E110, _:L111, _:P112, _:R113, _:F114, _:M115, _:N116, _:Y117, _:L119, _:N120, _:N121, _:T122, _:K123, _:G418, _:K419, _:T420	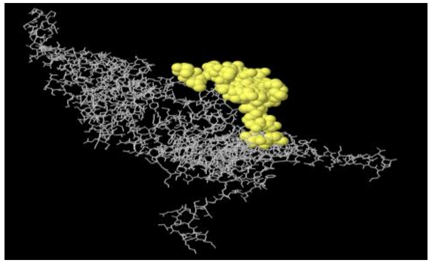
:S259, _:N262, _:D263, _:M264, _:P265, _:I266, _:T267, _:N268, _:D269, _:K271	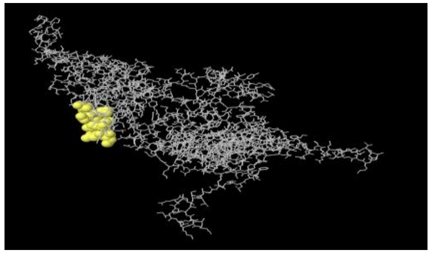
_:N426, _:K427, _:D428, _:R429, _:G430, _:I431, _:I432, _:G446, _:V447, _:D448	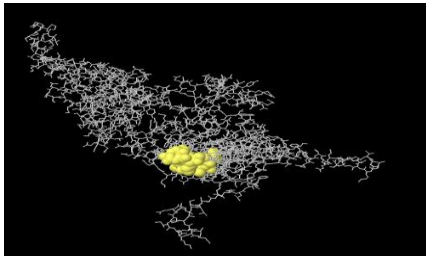
:A346, _:G347, _:S348, _:P376, _:S377, _:E378, _:L381, _:I384, _:D385, _:I386, _:N388, _:P389, _:K390	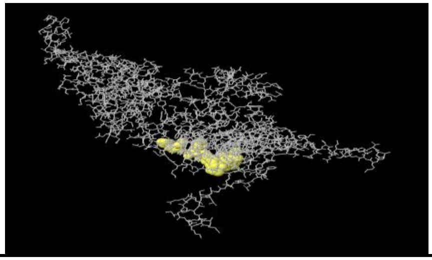

C69, _:N70, _:G71, _:T72, _:D73, _:A74, _:K75, _:V76, _:K77	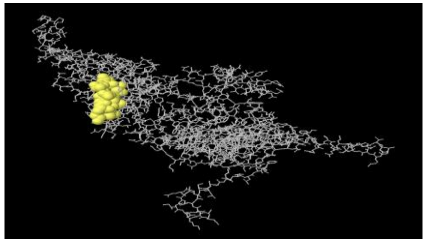
:T323, _:T324, _:N325, _:T326, _:K327, _:E328, _:G329, _:S330, _:N331, _:K399	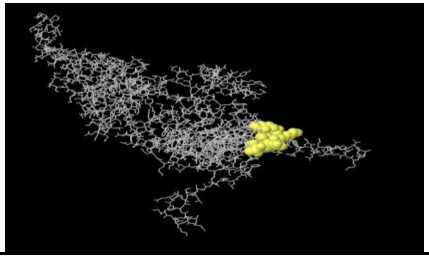

Table 7. Codon optimized nucleotide sequence of MEV construct for cloning in *E. coli* strain K12. Red color bold sequence at 5` site (N-terminal) is representing HindIII restriction enzyme site, while green color bold sequence at 3` site (C-terminal) is representing BamHI restriction enzyme site.

**AAGCTT**GGTATCATCAACACCCTGCAGAAATACTACTGCCGTGTTCGTGGTGGTCGTTGCGCTGTTCTGTCTTGCCTGCCGAAAGAAGAACAGATCG<br>GTAAATGCTCTACCCGTGGTCGTAAATGCTGCCGTCGTAAAAAAGAAGCTGCTGCTAAAAAATCTATCGCTCAGATCACCCTGTCTATCCTGGCTGC<br>TGCTTACTACCTGACCCAGAACCCGCAGCTGGGTATCTCTTTCGCTGCTTACACCACCAAACAGCGTCAGAACAAACCGCCGAACAAAGCTGCTTA<br>CTGCTCTATCTGCTCTAACAACCCGACCTGCTGGGCTGCTGCTTACTGCTCTAACAACCCGACCTGCTGGGCTATCTGCAAAGCTGCTTACTCTTCTG<br>AAGGTAACATCTCTCCGTCTCAGGTTTACGCTGCTTACGAAACCGTTATCGAATTCCAGCAGAAAAACAACCGTGCTGCTTACGACACCCCGTGCTG<br>GAAACTGCACACCTCTCCGCTGGCTGCTTACTCTGTTTCTTTCTTCCCGCTGGCTGAAACCTGCAAAGCTGCTTACTTCTTCCCGCTGGCTGAAACCTG<br>CAAAGTTCAGTCTGCTGCTTACTCTCTGTACGTTAAAGGTGAACCGATCATCAACTTCGCTGCTTACTGCAAAGCTCGTTCTACCCCGGTTACCCTGT<br>CTAAAGCTGCTTACCGTTCTACCCCGGTTACCCTGTCTAAAGACCAGCTGGGTCCGGGTCCGGGTGCTATCATCTTCATCGCTTCTGCTAACAACAA<br>AGTTACCCTGACCGGTCCGGGTCCGGGTATCATCTTCATCGCTTCTGCTAACAACAAAGTTACCCTGACCACCG**GATCC**

© 2020 by the authors. Submitted for possible open access publication under the terms and conditions of the Creative Commons Attribution (CC BY) license (http://creativecommons.org/licenses/by/4.0/).
